# A SF_5_ Derivative of Triphenylphosphine as an Electron-Poor Ligand Precursor for Rh and Ir Complexes

**DOI:** 10.3390/molecules25173977

**Published:** 2020-09-01

**Authors:** Maria Talavera, Silke Hinze, Thomas Braun, Reik Laubenstein, Roy Herrmann

**Affiliations:** Department of Chemistry, Humboldt–Universität zu Berlin, Brook-Taylor-Str. 2, 12489 Berlin, Germany; talaverm@hu-berlin.de (M.T.); silkehinze@gmx.de (S.H.); reik.laubenstein@gmx.de (R.L.); roy.herrmann@gmx.de (R.H.)

**Keywords:** fluorosulfanyl group, fluorinated ligands, phosphines, rhodium, iridium

## Abstract

The synthesis of the triarylphosphine, P(*p*-C_6_H_4_SF_5_)_3_ containing a SF_5_ group, has been achieved. The experimental and theoretical studies showed that P(*p*-C_6_H_4_SF_5_)_3_ is a weaker σ-donor when compared with other substituted triarylphosphines, which is consistent with the electron-withdrawing effect of the SF_5_ moiety. The studies also revealed a moderate air stability of the phosphine. The σ-donor capabilities of P(*p*-C_6_H_4_SF_5_)_3_ were estimated from the phosphorus-selenium coupling constant in SeP(*p*-C_6_H_4_SF_5_)_3_ and by DFT calculations. The behavior of P(*p*-C_6_H_4_SF_5_)_3_ as ligand has been investigated by the synthesis of the iridium and rhodium complexes [MCl(COD){P(*p*-C_6_H_4_SF_5_)_3_}], [MCl(CO)_2_{P(*p*-C_6_H_4_SF_5_)_3_}_2_] (M = Ir, Rh), or [Rh(*µ*-Cl)(COE){P(*p*-C_6_H_4_SF_5_)_3_}]_2_, and the molecular structures of [IrCl(COD){P(*p*-C_6_H_4_SF_5_)_3_}] and [Rh(*µ*-Cl)(COE){P(*p*-C_6_H_4_SF_5_)_3_}]_2_ were determined by single X-ray diffraction. The structures revealed a slightly larger cone angle for P(*p*-C_6_H_4_SF_5_)_3_ when compared to other *para*-substituted triarylphosphines.

## 1. Introduction

Phosphines are one of the most important and widely used ligands in homogeneous catalysis and organometallic chemistry due to the extensive options to modify their electronic and steric properties by varying their substitution pattern [1,2,3,4]. Phosphines are σ-donor and π-acceptor ligands and the choice of the phosphorus-bound entities can effectively tune their electronic characteristics. Thus, the electronic, but also steric properties of triarylphosphines are highly dependent on the employed aryl groups [1,2,3,4].

Triarylphosphines bearing fluorine atoms or fluorinated substituents are characterized by a higher steric demand and a lower basicity than the non-halogenated counterparts which might lead to changes in the reactivity [5]. Thus, complexes bearing fluorinated triarylphosphines have been widely investigated over the last years [5,6,7,8,9,10,11,12,13]. In particular, arylphosphines containing a CF_3_ group have been studied for catalytic processes such as hydroformylation [14], C-C coupling [13,15,16,17] as well as hydroalkylation reactions [18]. They present an alternative to fluoroarylphosphines with respect to the increased electron-withdrawing properties of the phosphine.

Another alternative and fascinating chemically and thermally rather stable group is the SF_5_ group [19,20], which displays a large σ-withdrawing nature (*F* = 0.56) and π-withdrawing ability (*R* = 0.12) based on the Swann-Lupton constants. In addition, phenyl groups with SF_5_ moieties at both *meta* and *para* positions exhibit a stronger electron-withdrawing effect according to Hammett constants (σ_m_ = 0.61, σ_p_ = 0.68), when compared with a fluorine substituent (*F* = 0.45, *R* = −0.39, σ_m_ = 0.34, σ_p_ = 0.06) or the trifluoromethyl group (*F* = 0.38, *R* = 0.16, σ_m_ = 0.43, σ_p_ = 0.54) [21]. Despite the increasing interest on SF_5_-containing compounds described in literature and their applications as building blocks in many fields such as agrochemical, medicinal, or materials chemistry [22,23,24,25,26,27,28,29,30], SF_5_ derivatized ligands in transition metal complexes are still rare [31,32,33,34,35,36,37,38,39,40,41]. Examples mainly focus on C^N cyclometalated ligands which contain the SF_5_ moiety in their phenyl rings in order to stabilize iridium(III) [34,35,36,37] or platinum(II) [38] complexes with optoelectronic properties.

Herein, we present the synthesis and characterization of a SF_5_ derivative of triphenylphosphine P(*p*-C_6_H_4_SF_5_)_3_ (**1**). Its air stability and electronic properties have been estimated and the derivatives O=P(*p*-C_6_H_4_SF_5_)_3_ (**2**) and Se=P(*p*-C_6_H_4_SF_5_)_3_ (**3**) were prepared. In addition, the rhodium and iridium complexes [MCl(COD){P(*p*-C_6_H_4_SF_5_)_3_}] (M = Ir (**4**), Rh (**5**)), [MCl(CO)_2_{P(*p*-C_6_H_4_SF_5_)_3_}_2_] (M = Ir (**8**), Rh (**9**)) or [Rh(*µ*-Cl)(COE){P(*p*-C_6_H_4_SF_5_)_3_}]_2_ (**6**) were synthesized.

## 2. Results and Discussion

### 2.1. Synthesis and Air-Stability of P(p-C_6_H_4_SF_5_)_3_ (1)

**Scheme 1:**
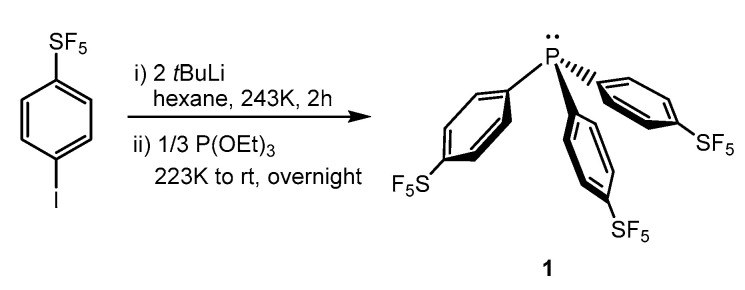
Synthesis of the phosphine **1**.

Treatment of 4-iodophenylsulfur pentafluoride with an excess of *t*BuLi and a subsequent reaction with triethylphosphite afforded the tris(*p*-pentafluorosulfanylphenyl)phosphine (**1**) in 39% isolated yield (Scheme 1). Compound **1** shows in the ^19^F NMR spectrum the characteristic signal pattern for the SF_5_ moiety [42], a doublet at 62.6 ppm corresponding to the four equatorial equivalent fluorine atoms and a pentet at 83.7 ppm for the axial fluorine with coupling constants of 150 Hz. The ^31^P{^1^H} NMR spectrum shows a singlet at −7.8 ppm, whereas the GC/MS gave a mass peak of *m/z* 640.

**Figure 1:**
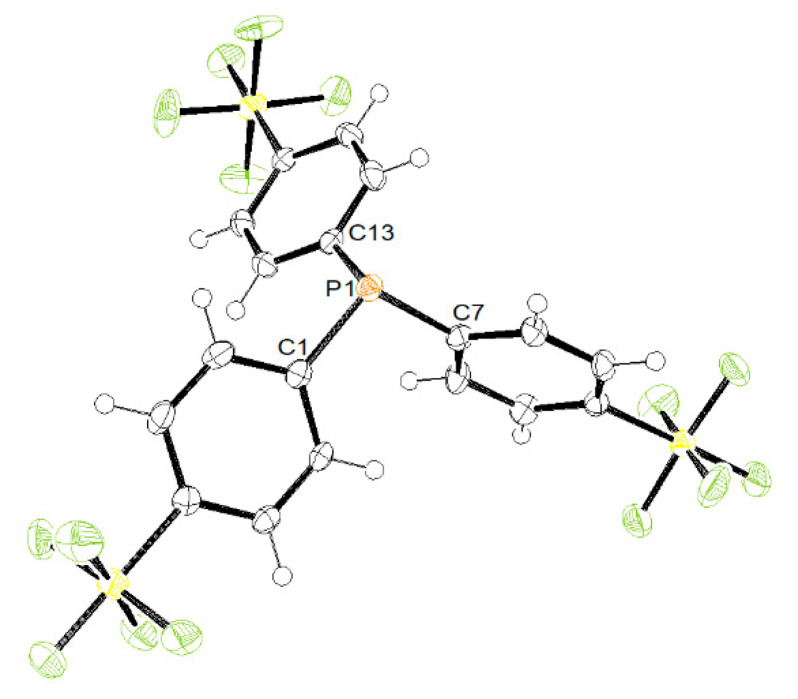
ORTEP representation of **1** with thermal ellipsoids drawn at 50% probability level. The C_6_H_6_ molecule in the asymmetric unit has been omitted for clarity. Selected bond lengths [Å] and bond angles [deg]: P1–C1 1.829(2), P1–C7 1.833(2), P1–C13 1.831(2), C1–P1–C7 103.21(10), C7–P1–C13 99.18(10), C13–P1–C1 103.08(10).

Single crystals of compound **1**, which were suitable for X-ray crystallography were obtained from concentrated solutions in benzene. The molecular structure is shown in Figure 1. Compound **1** shows a trigonal pyramidal arrangement at the phosphorus atom when taking the electron lone-pair into account. The C–P–C angles are comparable to the ones reported for triphenylphosphine, where only one angle is slightly larger (101.72(1)° for PPh_3_ vs. 99.18(10)° for **1**) than the others [43].

**Scheme 2:**
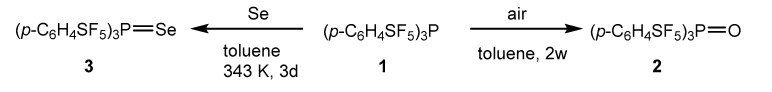
Formation of the phosphine oxide **2** and the phosphine selenide **3**.

In order to test the air-stability of the phosphine **1**, a solution of **1** in toluene-d_8_ was left under air for 2 weeks. Seventy-eight percent of the phosphine **1** was then converted into phosphine oxide **2** (Scheme 2). In the solid state 41% of conversion was achieved after a month of air exposure. These data indicate a higher stability of **1** towards air when compared with the CF_3_ analogue, which is fully oxidized in the solid state after one month [44]. ^31^P{^1^H} NMR data of the solution as well as liquid injection field desorption/ionization mass spectrometry (LIFDI-MS) confirm the formation of the oxide with a shift of the signal in the ^31^P{^1^H} NMR to lower field (*δ* = 21.09 ppm) and a mass peak of *m/z* 656.

**Figure 2:**
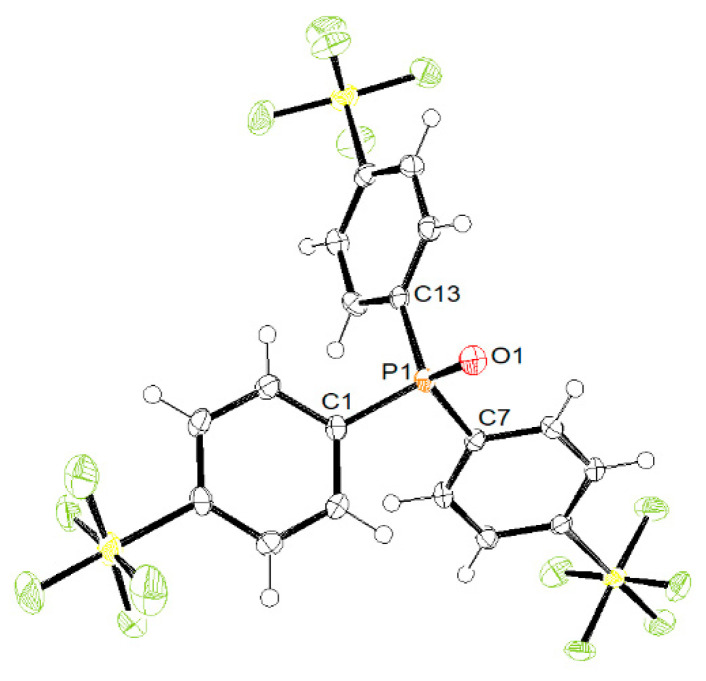
ORTEP representation of **2** with thermal ellipsoids drawn at 50% probability level. The two C_6_H_6_ molecules contained in the asymmetric unit have been omitted for clarity. Selected bond lengths [Å] and bond angles [deg]: P1–O1 1.4867(14), P1–C1 1.8086(19), P1–C7 1.8086(19), P1–C13 1.8019(19), C1–P1–C7 107.19(8), C7–P1–C13 106.28(8), C13–P1–C1 106.35(9), O1–P1–C1 113.23(8), O1–P1–C7 111.35(8), O1–P1–C13 112.02(8).

Crystals of **2** suitable for X-ray diffraction analysis were obtained from the reaction solution (Figure 2). The compound shows the expected tetrahedral arrangement with a C–P–C mean angle of 106.6°, a O–P–C mean angle of 112.2° and the P–O bond length of 1.4867(14) Å. All of the data are consistent with these of other reported triarylphosphine oxides derivatives [45].

In order to further compare the air-stability of phosphine **1** with other triarylphosphines, DFT calculations were performed. It has been suggested previously that the steric demand of the phosphines, but also the SOMO energy of radical cations of phosphines can be correlated with their air-stability [46]. Thus, a radical cation with a SOMO at lower energy would be more prone to react with dioxygen generating the corresponding phosphine oxide [46]. Therefore, the energy of the SOMO of different triarylphosphines radical cations was calculated using the CAM-B3LYP functional (Table 1). The phosphine **1** has a SOMO energy of −12.22 eV, which is lower than the energy of other electron-withdrawing triarylphosphines, and lower than the one of the air-stable triphenylphoshine (−11.11 eV). The data are in accordance with the observed moderate air sensitivity of phosphine **1**.

### 2.2. Estimation of the Donor Properties

Different methods have been reported to determine the electronic properties of phosphines. The σ-donor ability increases when the s-character of the lone pair of the phosphine decreases, which is associated with a higher energy level of the HOMO [47,48]. Thus, DFT studies have been performed in order to calculate the energy level of the HOMO of compound **1** and compare it with other triarylphosphines which were also calculated. The data indicate that compound **1** (−8.69 eV) is a less pronounced σ-donor than most of the calculated phosphines (Table 2). It is worth noting that P(*p*-C_6_H_4_CF_3_)_3_ (−8.17 eV) seems, according to this data, to be a better σ-donor than **1**, whereas P(*m*-C_6_H_3_(CF_3_)_2_)_3_ is a weaker σ-donor (−8.78 eV).

The HOMO energy level and, therefore, the s-character of the lone pair is also related to the phosphorus-selenium coupling constant of the corresponding selenide. This has been commonly used to experimentally estimate the σ-donor abilities of a broad range of phosphines [49,50,51]. Thus, for more electron-withdrawing substituents a larger coupling is expected [51]. Taking this into account, phosphine **1** was reacted with selenium and after 3d, a full conversion to SeP(*p*-C_6_H_4_SF_5_)_3_ (**3**) was observed (Scheme 2). The ^31^P{^1^H} NMR spectrum shows a singlet at *δ* 32.5 ppm with selenium satellites and a phosphorus-selenium coupling constant of 792 Hz. Correspondingly, the resonance in the ^77^Se NMR spectrum at −273.3 ppm appears as a doublet with the same coupling constant. Among the data for the phosphines shown in Table 2 only P(*m*-C_6_H_3_(CF_3_)_2_)_3_ shows a larger coupling constant of 802 Hz, which is consistent with the lower HOMO energy values.

Another common method to determine the electronic properties of ligands is the calculation of the Tolman’s electronic parameter TEP [52]. This method consists in the analysis of the frequency of the A_1_ carbonyl vibration mode of [Ni(CO)_3_L] complexes, which will decrease due to the back-donation into the CO π* orbitals when the ligand L is a better donor. While Tolman experimentally determined the parameter for a broad range of phosphines [52], DFT studies have demonstrated the correlation between the calculated and experimental values for different ligands L [53,54]. Thus, the calculated values determined in this work correlate well with the experimentally obtained for PPh_3_ and P(*p*-C_6_H_4_Me)_3_ (Δ_exp-calc_ ≈ 5 cm^−1^) (Table 2) [52]. The data also indicate that the phosphine **1** might be a slightly more electron-withdrawing phosphine than P(*p*-C_6_H_4_CF_3_)_3_, but somewhat weaker than P(*m*-C_6_H_3_(CF_3_)_2_)_3_, although the calculated values are very close.

### 2.3. Synthesis of Iridium and Rhodium Complexes

**Scheme 3:**
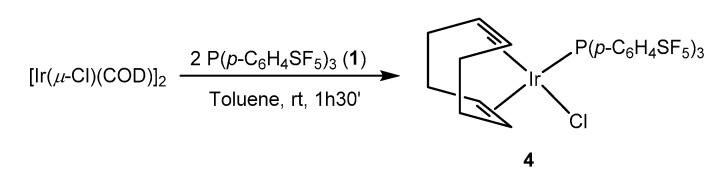
Synthesis of the iridium(I) complex **4**.

Treatment of the binuclear iridium complex [Ir(*µ*-Cl)(COD)]_2_ (COD = 1,5-cyclooctadiene) with two equivalents of the phosphine **1** in toluene yielded the iridium(I) complex [IrCl(COD){P(*p*-C_6_H_4_SF_5_)_3_}] (**4**) (Scheme 3). The same reactivity was reported for other phosphines such as P(*p*-C_6_H_4_CF_3_)_3_ [56]. The structure of **4** is supported by the ^31^P{^1^H} NMR spectrum, which shows a resonance at *δ* 21.7 ppm and the ^1^H NMR spectrum with two resonances at *δ* 5.64 and 2.45 ppm corresponding to the olefinic protons of the COD ligand in a *trans* arrangement to the phosphine and the chlorido ligands. The LIFDI mass spectrometry reveals a mass peak of *m/z* 976.

**Scheme 4:**
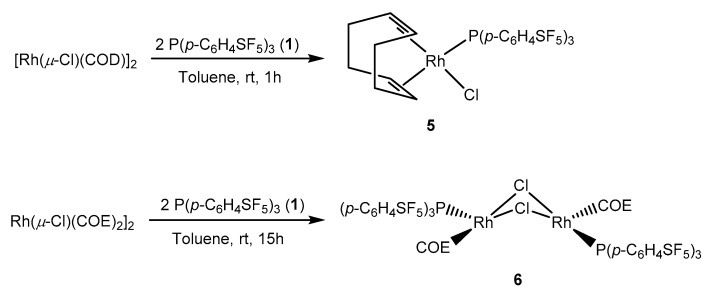
Synthesis of the rhodium(I) complexes **5** and **6**.

Analogously, [Rh(*µ*-Cl)(COD)]_2_ reacted with two equivalents of the phosphine **1** affording the rhodium(I) complex [RhCl(COD){P(*p*-C_6_H_4_SF_5_)_3_}] (**5**) (Scheme 4). The ^31^P{^1^H} NMR spectrum of **5** depicts a doublet at *δ* 31.5 ppm with a rhodium-phosphorus coupling constant of 155.9 Hz. The coupling constant of **5** is 1.8 Hz higher than the one for [RhCl(COD){P(*p*-C_6_H_4_CF_3_)_3_}] (^1^*J*_P-Rh_ = 154.1 Hz) [57]. As for the iridium complex **4**, ^1^H NMR spectrum of complex **5** shows two resonances for the olefinic protons of the cyclooctadiene ligand at *δ* 5.67 and 3.12 ppm which are also consistent with the CF_3_ derivative [57].

**Scheme 5:**
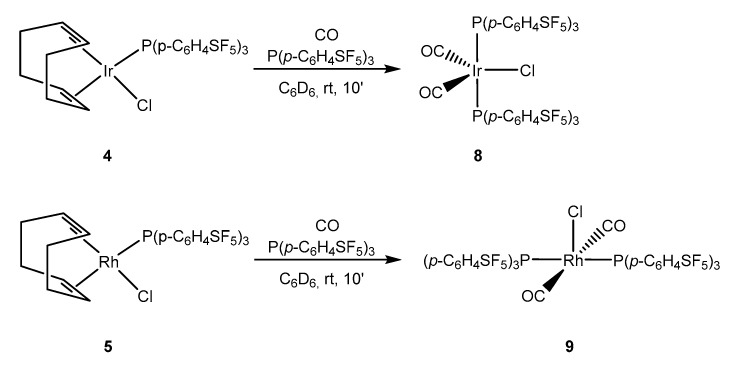
Reactivity of the complexes **4** and **5** towards CO in presence of phosphine **1**.

In contrast, when [Rh(*µ*-Cl)(COE)_2_]_2_ (COE = *cis*-cyclooctene) was used as binuclear starting compound, the reaction with two equivalents of phosphine **1** selectively provided *trans*-[Rh(*µ*-Cl)(COE){P(*p*-C_6_H_4_SF_5_)_3_}]_2_ (**6**) (Scheme 5). The dimeric nature of the product is supported by the ^31^P{^1^H} NMR data revealing a signal at *δ* 55.5 ppm with an increased value of the rhodium-phosphorus coupling constant (^1^*J*_P-Rh_ = 194.5 Hz). This coupling constant value is slightly larger than for other *trans*-[Rh(*µ*-Cl)(COE)(L)]_2_ complexes, for which L is a σ-donor phosphine (^1^*J*_P-Rh_ = 183–188Hz) [58,59].

### 2.4. Steric Properties of 1

Steric properties of phosphines is another important feature when studying a ligand. The Tolman cone angle [52] of a phosphine is a widely used parameter to describe the steric effects in phosphines and can be estimated not only from molecular models but also from molecular structures determined by X-ray crystallography [60].

**Figure 3:**
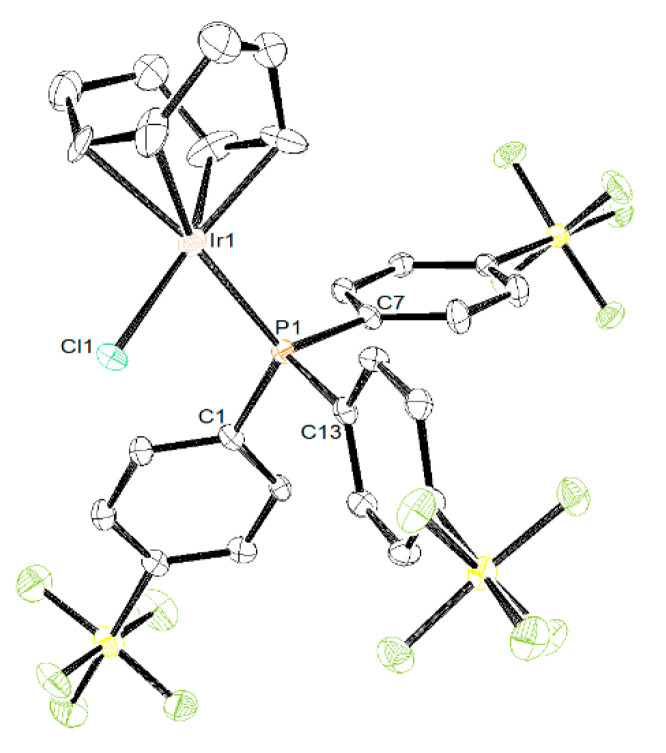
ORTEP representation of **4** with thermal ellipsoids drawn at 50% probability level. Hydrogen atoms and the additional C_6_H_6_ molecule in the unit cell have been omitted for clarity. Note that some disorder is observed in COD ligand. Selected bond lengths [Å] and bond angles [deg]: Ir1–P1 2.2888(12), Ir1–Cl1 2.4070(10), P1–C1 1.831(5), P1–C7 1.834(5), P1–C13 1.838(5), Cl1–Ir1–P1 90.27(4), Ir1–P1–C1 118.23(15), Ir1–P1–C7 118.61(15), Ir1–P1–C13 108.27(16), C1–P1–C7 100.7(2), C7–P1–C13 104.2(2), C13–P1–C1 105.3(2).

**Figure 4:**
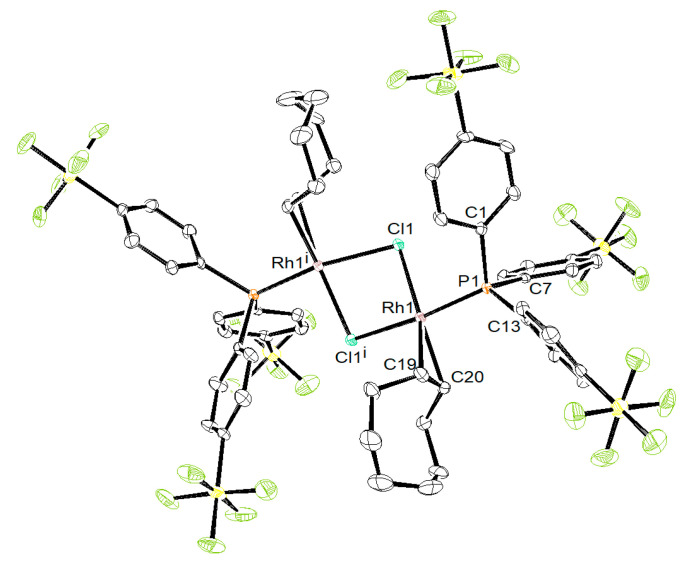
ORTEP representation of **6** with thermal ellipsoids drawn at 50% probability level. Hydrogen atoms and the C_6_H_6_ molecule in the unit cell have been omitted for clarity. Selected bond lengths [Å] and bond angles [deg]: Rh1–P1 2.1930(11), Rh1–Cl1 2.3911(11), Rh1–Cl1^i^ 2.4368(11), Rh1–C19 2.120(4), Rh1–C20 2.131(4), P1–C1 1.827(4), P1–C7 1.842(4), P1–C13 1.825(4), Cl1–Rh1–Cl1^i^ 80.57(4), Cl1–Rh1–P1 89.66(4), Cl1^i^–Rh1–P1 166.80(4), P1–Rh1–C19 94.48(12), P1–Rh1–C20 93.38(12), Rh1–P1–C1 113.38(14), Rh1–P1–C7 111.20(14), Rh1–P1–C13 120.94(14), C1–P1–C7 103.73(19), C7–P1–C13 103.51(19), C13–P1–C1 102.26(19).

The molecular structures of the complexes **4** and **6** were obtained by X-ray diffraction analysis from concentrated C_6_H_6_ solutions (Figure 3 and Figure 4). In the case of complex **4**, the iridium center displays a square planar coordination geometry when considering COD as a bidentate ligand with an Ir–P bond distance of 2.2888(12) Å, which is slightly shorter than the corresponding separation in [IrCl(COD){P(C_6_H_5_)_3_}] (2.3172(9) Å) [61].

The Tolman cone angle of compound **1** can be estimated to be 150.8° as the average value for the complexes **4** and **6**, and by considering van der Waals radii of 1.20 Å and 1.47 Å for the H and F nuclei, respectively [62]. For the calculations, the algorithm reported by Müller and Mingos was used and the metal-phosphorus distances were fixed to 2.28 Å [60]. The obtained value is slightly larger than for other triarylphosphines with a substituent at the *para* position P(*p*-C_6_H_4_X)_3_ (X = NMe_2_, Me, OMe, F, CF_3_), all of which have a cone angle of 145° [52,63].

### 2.5. Reactivity of Complexes 4 and 5 towards CO

Bubbling of CO into a dichloromethane solution of [IrCl(COD){P(*p*-C_6_H_4_SF_5_)_3_}] (**4**) resulted in a partial conversion of **4** to yield a complex, for which we suggest the structure [IrCl(CO)(COD){P(*p*-C_6_H_4_SF_5_)_3_}] (**7**). However, when a degassed solution was treated with CO gas, full conversion of complex **4** was observed and a dark unidentified precipitate was formed. In the solution, the formation of a complex containing both COD and CO ligands without the presence of phosphine **1** was detected. Unfortunately, no further identification of this complex was possible. In addition, two products bearing the phosphine ligand **1** in a 1:1 ratio were obtained. Thus complex [IrCl(CO)(COD){P(*p*-C_6_H_4_SF_5_)_3_}] (**7**) was formed together with a second complex, the analytical data of which are consistent with the structure [IrCl(CO)_2_{P(*p*-C_6_H_4_SF_5_)_3_}_2_] (**8**). To support the structural assignments further, ^13^C labeled carbon monoxide was reacted with a solution of complex **4** to give **7′** and **8′**. However, the formation of the unknown complex as well as the mixture of products was avoided and a full conversion of **4** into complexes **8** or **8′** was achieved, when the reaction was performed in presence of one equivalent of phosphine **1** (Scheme 5).

Complexes **7** and **8** show singlet resonances for the phosphine ligands at *δ* 3.65 and 0.84 ppm, respectively, in the ^31^P{^1^H} NMR spectrum. In addition, the signals for the olefinic protons of COD in complex **7** appear at *δ* 4.25 and 3.91 ppm, which would correspond to the protons *trans* to the CO and Cl ligands, respectively, suggesting that the phosphine ligand is in a apical position. The ^31^P{^1^H} NMR spectrum for the mixture of complexes **7′** and **8′** showed the two resonances at *δ* 3.52 and 0.84 ppm as a doublet (^2^*J*_P-C_ = 13.7 Hz) and a triplet (^2^*J*_P-C_ = 13.5 Hz), respectively. Note that the values of the carbon-phosphorus coupling constants are consistent with *cis* arrangements [64,65]. On the other hand, in the ^13^C{^1^H} NMR spectrum a doublet (*δ* = 175.6 ppm, complex **7′**) and a triplet (*δ* = 179.6 ppm, complex **8′**) with similar coupling constants are observed for the carbonyl ligands.

The trigonal bipyramid proposed for complex **8** is supported by the IR data in the solid state. The IR spectrum of complex **8** shows two bands at 1940 and 1986 cm^−1^ for the symmetric and the asymmetric stretching bands of the CO ligands, which shift to 1896 and 1933 cm^−1^ for **8′** suggesting that the CO ligands are in an equatorial position (see Figure S39). This data for **8** are in the same range as the ones observed for [IrCl(CO)_2_(PPh_3_)_2_] [66].

For comparison, [RhCl(COD){P(*p*-C_6_H_4_SF_5_)_3_}] (**5**) was also reacted with CO or ^13^CO in the presence of one equivalent of phosphine **1** to give complexes, for which we suggest the structures *trans,trans-*[RhCl(CO)_2_{P(*p*-C_6_H_4_SF_5_)_3_}_2_] (**9**) and *trans,trans-*[RhCl(^13^CO)_2_{P(*p*-C_6_H_4_SF_5_)_3_}_2_] (**9′**) (Scheme 5). The NMR data of **9** showed a broad band in the ^31^P{^1^H} NMR spectrum at room temperature at *δ* 28.6 ppm. When the sample was measured at −70 °C, the coupling to rhodium was observed (^1^*J*_P-Rh_ = 130 Hz). As also found for the PPh_3_ analogue [67], the coupling to carbon in the ^13^C labeled complex was not observed even not at −70 °C. The ^13^C{^1^H} NMR spectrum revealed a Rh-C coupling constant of 72 Hz in the doublet at 186.5 ppm. Finally, in the IR spectrum, one unique band was observed for the CO ligands at 1992 and 1945 cm^−1^ for the complexes **9** and **9′**, respectively (see Figure S40). This is consistent with data for [RhCl(CO)_2_(PPh_3_)_2_], where only one stretching band was observed for CO at 1990 cm^−1^ and a square pyramidal structure with the chlorido ligand in the apical position as the most probable structure was proposed [67].

## 3. Materials and Methods

### 3.1. General Procedures, Methods and Materials

All experiments were carried out under an atmosphere of argon by Schlenk techniques. Solvents were dried by the usual procedures [68] and, prior to use, distilled under argon. The rhodium and iridium complexes [Rh(*µ*-Cl)(COE)_2_]_2_ and [Ir(*µ*-Cl)(COD)]_2_ were prepared as described in the literature [69,70]. All reagents were obtained from commercial sources. Unless stated, NMR spectra were recorded at room temperature on a Bruker DPX 300 (Bruker BioSpin, Rheinstetten, Germany,) or a Bruker Avance 300 spectrometer (Bruker BioSpin, Rheinstetten, Germany). ^1^H and ^13^C{^1^H} signals are referred to residual solvent signals, those of ^31^P{^1^H} to external 85% H_3_PO_4_, the ^19^F NMR spectra to external CFCl_3_ and the ^77^Se NMR spectra to external SePh_2_ (*δ* = 414 ppm). ^1^H and ^13^C{^1^H} NMR signal assignments were confirmed by ^1^H{^31^P}, ^1^H,^1^H COSY, ^1^H,^13^C HMQC and ^1^H,^13^C HMBC NMR experiments. Mass spectra were measured with a Micromass Q–Tof–2 instrument equipped with a Linden LIFDI source (Linden CMS GmbH, Weyhe, Germany). GC/MS analyses were performed with an Agilent 6890N gas–phase chromatograph (Shimadzu, Berlin, Germany) equipped with an Agilent 5973 Network mass selective detector at 70eV. Infrared spectra were recorded with the Platinum ATR module of a Bruker FT-IR Alpha II spectrometer (Bruker Optics, Leipzig, Germany) equipped with an ATR unit (diamond). NMR spectra are included as Supplementary Material (Figures S1–S38).

### 3.2. Synthesis of Tris-(p-pentafluorosulfanylphenyl)phosphine (1)

4-iodophenylsulfur pentafluoride (250 mg, 0.76 mmol) was dissolved in 10 mL of hexane at 243 K. Then, two equivalents of *tert*–buthyllithium (1.7 M in pentanes, 1.52 mmol, 0.9 mL) was added dropwise to the solution and the reaction mixture was stirred for 2 h at 243 K. Afterwards, the mixture was cooled down to 223 K and triethylphosphite (0.25 mmol, 45 µL) was added slowly. The mixture was stirred while warming up overnight. The volatiles were removed under vacuum, toluene (2 × 10 mL) was added and the product extracted. The solvent was removed from the extract and the beige solid obtained dried under vacuum. Yield: 190 mg (39%).

GC–MS (toluene): Calculated (*m/z*) for [M]: 640.43; found: 640. ^31^P{^1^H} NMR (121.5 MHz, C_6_D_6_): δ = −7.8 (s) ppm. ^1^H NMR (300.1 MHz, C_6_D_6_): δ = 7.26 (dm, ^3^*J*(H,H) = 8.7 Hz, 6H, *m*–C*H*); 6.74 (dd, ^3^*J*(H,H) = 8.6, ^2^*J*(H,P) = 6.8 Hz, 6H, *o*–C*H*) ppm. ^19^F NMR (282.4 MHz, C_6_D_6_): δ = 83.7 (p, ^2^*J*(F,F) = 150 Hz, 1F, S*F*_5_); 62.6 (d, ^2^*J*(F,F) = 150 Hz, 4F, S*F*_5_) ppm. ^13^C{^1^H} NMR (75.4 MHz, CD_2_Cl_2_): 155.5–154.1 (m, *C_q_*–SF_5_); 140.6 (d, ^1^*J*(C,P) = 15.4 Hz, P–*C*_q_); 134.5 (d, ^2^*J*(C,P) = 20.9 Hz, *o*–*C*H); 126.8 (dp, ^3^*J*(C,P) = 6.8 Hz, ^3^*J*(C,F_eq_) = 4.3 Hz, *m*–*C*H) ppm.

### 3.3. Formation of Tris-(p-pentafluorosulfanylphenyl)phosphine oxide (2)

Method a: Tris-(*p*-pentafluorosulfanylphenyl)phosphine **1** (20 mg, 0.03 mmol) was dissolved in toluene-*d^8^* (0.4 mL) and the solution was exposed under air. The conversion was followed by NMR spectroscopy, and after 2 weeks 78% conversion was observed.

Method b: Tris-(*p*-pentafluorosulfanylphenyl)phosphine **1** (20 mg, 0.03 mmol) was exposed under air in an open vial. After 1 month, a 41% conversion to compound **2** was found.

LIFDI (toluene-*d^8^*): Calculated (*m/z*) for [M]: 656.4; found: 656. ^31^P{^1^H} NMR (121.5 MHz, toluene–*d_8_*): δ = 21.1 (s) ppm. ^1^H NMR (300.1 MHz, toluene–*d^8^*): δ = 7.30 (dd, ^3^*J*(H,H) = 8.3; ^3^*J*(H,P) = 2.2 Hz, 6H, *m*-C*H*); 7.21 (dd, ^2^*J*(H,P) = 10.9, ^3^*J*(H,H) = 8.3 Hz, 6H, *o*-C*H*) ppm. ^19^F NMR (282.4 MHz, toluene-*d^8^*): δ = 82.7 (p, ^2^*J*(F,F) = 150 Hz, 1F, S*F*_5_); 62.3 (d, ^2^*J*(F,F) = 150 Hz, 4F, S*F*_5_) ppm.

### 3.4. Synthesis of Tris-(p-pentafluorosulfanylphenyl)phosphine Selenide (3)

Tris-(*p*-pentafluorosulfanylphenyl)phosphine **1** (50 mg, 0.08 mmol) was dissolved in toluene (10 mL) and one equivalent of selenium (6 mg, 0.08 mmol) was added. Then, the reaction mixture was stirred at 343 K for 3 days. The reaction solution was filtered and the volatiles were removed from the filtrate. The dark solid was dried in vacuum. Yield: 54 mg (94%).

LIFDI (toluene-*d^8^*): Calculated (*m/z*) for [M]: 719.39; found: 719. ^31^P{^1^H} NMR (202.4 MHz, toluene–*d_8_*): δ = 32.5 (s + sat, ^1^*J*(P,Se) = 791.8 Hz) ppm. ^77^Se NMR (95.4 MHz, toluene-*d^8^*): δ = −273.3 (d, ^1^*J*(Se,P) = 792.3 Hz) ppm. ^1^H NMR (500.1 MHz, toluene-*d^8^*): δ = 7.27 (dd, ^2^*J*(H,P) = 12.8; ^3^*J*(H,H) = 8.7 Hz, 6H, *o*-C*H*); 7.20 (dd, ^3^*J*(H,H) = 8.8, ^3^*J*(H,P) = 2.2 Hz, 6H, *m*-C*H*) ppm. ^19^F NMR (470.6 MHz, toluene-*d_8_*): δ = 82.4 (p, ^2^*J*(F,F) = 150 Hz, 1F, S*F*_5_); 62.3 (d, ^2^*J*(F,F) = 150 Hz, 4F, S*F*_5_) ppm.

### 3.5. Synthesis of [IrCl(COD){P(p-C_6_H_4_SF_5_)_3_}] (4)

[Ir(*µ*-Cl)(COD)]_2_ (100 mg, 0.15 mmol) (COD = cyclooctadiene) was dissolved in toluene (7 mL) and a solution of P(*p*-C_6_H_4_SF_5_)_3_ (**1**) (192 mg, 0.30 mmol) in 5 mL of toluene was added slowly. After stirring for 1 h 30′, the volatiles were removed under vacuum and a dark red solid was obtained. The solid was washed with hexane (3 × 5 mL) and dried in vacuum. Yield: 117 mg (80%).

LIFDI-TOF-MS (toluene): Calculated (*m/z*) for [M]^+^: 976.28; found: 976. ^31^P{^1^H} NMR (121.5 MHz, C_6_D_6_): δ = 21.7 (s) ppm. ^1^H NMR (300.1 MHz, C_6_D_6_): δ = 7.41–7.23 (m, 12H, *Ph*); 5.64 (s br, 2H, =C*H trans* to P); 2.45 (s br, 2H, =C*H trans* to Cl); 2.16–1.84 (m, 2H, C*H_2_*); 1.82–1.57 (m, 2H, C*H_2_*); 1.54–1.29 (m, 2H, C*H_2_*); 1.17–0.85 (m, 2H, C*H_2_*) ppm. ^19^F NMR (282.4 MHz, C_6_D_6_): δ = 83.0 (p, ^2^*J*(F,F) = 150 Hz, 1F, S*F*_5_); 62.4 (d, ^2^*J*(F,F) = 150 Hz, 4F, S*F*_5_) ppm.

### 3.6. Synthesis of [RhCl(COD){P(p-C_6_H_4_SF_5_)_3_}] (5)

[Rh(*µ*-Cl)(COD)]_2_ (50 mg, 0.10 mmol) was dissolved in toluene (5 mL) and P(*p*-C_6_H_4_SF_5_)_3_ (**1**) (130 mg, 0.20 mmol) was added to the solution. After stirring for 1h15′, the volatiles were removed under vacuum and a yellow solid was obtained. The solid was washed with cold hexane (2 × 4 mL) and finally dried in vacuum. The NMR spectra of the yellow solid confirmed the formation of complex **5**. Yield: 162 mg (92%).

^31^P{^1^H} NMR (121.5 MHz, C_6_D_6_): δ = 31.5 (d, ^1^*J*(P,Rh) = 155.9 Hz) ppm. ^1^H NMR (300.1 MHz, C_6_D_6_): δ = 7.89–7.78 (m, 12H, *Ph*); 5.67 (s br, 2H, =C*H trans* to P); 3.12 (s br, 2H, =C*H trans* to Cl); 2.55–2.35 (m, 4H, C*H_2_*); 2.25–1.94 (m, 4H, C*H_2_*) ppm. ^19^F NMR (282.4 MHz, C_6_D_6_): δ = 82.33 (p, ^2^*J*(F,F) = 150 Hz, 1F, S*F*_5_); 61.89 (d, ^2^*J*(F,F) = 150 Hz, 4F, S*F*_5_) ppm. ^13^C{^1^H} NMR (75.4 MHz, CD_2_Cl_2_): 156.4–155.0 (m, *C_q_*–SF_5_); 135.7 (m, overlapped with CH signals, P–*C*_q_); 135.6 (d, ^2^*J*(C,P) = 12.8 Hz, *o*–*C*H); 126.4 (dp, ^3^*J*(C,P) = 9.6 Hz, ^3^*J*(C,F_eq_) = 4.8 Hz, *m*–*C*H); 108.7 (dd, ^1^*J*(C,Rh) = 12.0 Hz; ^2^*J*(C,P) = 7.2 Hz, =*C*H trans to P); 72.2 (d, ^1^*J*(C,Rh) = 12.8 Hz, =*C*H trans to Cl); 33.44, 33.43 and 29.2 (all s, *C*H_2_) ppm.

### 3.7. Synthesis of [Rh(µ-Cl)(COE){P(p-C_6_H_4_SF_5_)_3_}]_2_ (6)

[Rh(*µ*-Cl)(COE)_2_]_2_ (50 mg, 0.07 mmol) (COE = cyclooctene) was dissolved in toluene (7 mL) and a solution of P(*p*-C_6_H_4_SF_5_)_3_ (**1**) (90 mg, 0.14 mmol) in 5 mL of toluene was added slowly. Instantly, the solution turned red and after stirring for 1 day, the volatiles were removed under vacuum. The red solid obtained was dried in vacuum. Yield: 123 mg (99%)

^31^P{^1^H} NMR (121.5 MHz, C_6_D_6_): δ = 54.4 (d, ^1^*J*(P,Rh) = 194.5 Hz) ppm. ^1^H NMR (300.1 MHz, C_6_D_6_): δ = 7.39–7.28 (m, 6H, *m*-C*H*); 7.54-7.40 (m, 6H, *o*-C*H*); 2.66–2.20 (m, 6H, COE); 1.55–1.15 (m, 8H, COE) ppm. ^19^F NMR (282.4 MHz, C_6_D_6_): δ = 83.2 (p, ^2^*J*(F,F) = 150 Hz, 1F, S*F*_5_); 62.4 (d, ^2^*J*(F,F) = 150 Hz, 4F, S*F*_5_) ppm.

### 3.8. Reaction of [IrCl(COD){P(p-C_6_H_4_SF_5_)_3_}] (4) with CO. Formation of [IrCl(CO)(COD){P(p-C_6_H_4_SF_5_)_3_}] (7) and [IrCl(CO)_2_{P(p-C_6_H_4_SF_5_)_3_}_2_] (8)

In a Young NMR tube, a solution of [IrCl(COD){P(*p*-C_6_H_4_SF_5_)_3_}] (**4**) (25 mg, 0.03 mmol) in CD_2_Cl_2_ (0.4 mL) was cooled to 77 K, degassed and treated with CO. After 10′ the solution turned dark brown and a black precipitate was formed. The NMR analysis showed full conversion to yield the complexes **7** and **8** in a 1:1 ratio together with an unknown iridium complex bearing no phosphine ligand.

NMR data for **7**: ^31^P{^1^H} NMR (121.5 MHz, CD_2_Cl_2_): δ = 3.65 (s) ppm. ^1^H NMR (300.1 MHz, CD_2_Cl_2_): δ 7.90–7.82 (m, 12H, *Ph*); 4.25 (s br, 2H, =C*H trans* to CO); 3.91 (s br, 2H, =C*H trans* to Cl); 2.95 (s br, 4H, C*H_2_*); 2.79–2.53 (m, 2H, C*H_2_*); 2.06-1.83 (m, 2H, C*H_2_*) ppm. ^19^F NMR (282.4 MHz, CD_2_Cl_2_): δ = 82.0 (p, ^2^*J*(F,F) = 150 Hz, 1F, S*F*_5_); 61.9 (d, ^2^*J*(F,F) = 150 Hz, 4F, S*F*_5_) ppm.

When the reaction was performed with ^13^CO, the complexes **7′** and **8′** were obtained.

Selected NMR data for **7′**: ^31^P{^1^H} NMR (121.5 MHz, CD_2_Cl_2_): δ = 3.65 (d, ^2^*J*(P,C) = 13.7 Hz) ppm. ^13^C{^1^H} NMR (75.4 MHz, CD_2_Cl_2_): 176.0 (t, ^2^*J*(C,P) = 13.6 Hz, *^13^C*O); 135.6-135.2 (m, P–*C*_q_ and *o*–*C*H);127.1–126.6 (m, *m*–*C*H) ppm.

NMR data for the unknown complex: ^1^H NMR (300.1 MHz, CD_2_Cl_2_): δ 4.79 (s br, 4H, =C*H*); 2.79–2.53 (m, 8H, C*H_2_*) ppm. ^13^C{^1^H} NMR (75.4 MHz, CD_2_Cl_2_): 169.7 (s br, *C*O); 80.4 (s, =*C*H); 33.7 (s, *C*H_2_) ppm.

### 3.9. Independent Formation of [IrCl(CO)_2_{P(p-C_6_H_4_SF_5_)_3_}_2_] (8)

In a Young NMR tube, complex [IrCl(COD){P(*p*-C_6_H_4_SF_5_)_3_}] (**4**) (25 mg, 0.03 mmol) was dissolved in C_6_D_6_ (0.4 mL) and the phosphine **1** (19 mg, 0.03 mmol) was added. Then, the solution was cooled to 77 K, degassed and treated with CO. After 10 min, the solution turned orange and a yellow precipitate was formed. The solid was filtered off, washed with C_6_D_6_ (2 × 0.25 mL) and dried under vacuum. Yield: 34 mg (85%).

^31^P{^1^H} NMR (121.5 MHz, CD_2_Cl_2_): δ = 0.84 (s) ppm. ^1^H NMR (300.1 MHz, CD_2_Cl_2_): δ 7.92 (dm, ^3^*J*(H,H) = 8.6 Hz, *m*–C*H*); 7.79 (dd, ^3^*J*(H,H) = 8.6, ^2^*J*(H,P) = 5.8 Hz, *o*–C*H*) ppm. ^19^F NMR (282.4 MHz, CD_2_Cl_2_): δ = 81.7 (p, ^2^*J*(F,F) = 150 Hz, 1F, S*F*_5_); 61.8 (d, ^2^*J*(F,F) = 150 Hz, 4F, S*F*_5_) ppm. ^13^C{^1^H} NMR (75.4 MHz, CD_2_Cl_2_): 156.1 (p, ^1^*J*(C,P) = 18.5 Hz, *C_q_*–SF_5_); 135.0 (m, overlapped with CH signals, P–*C*_q_); 134.9 (d, ^2^*J*(C,P) = 12.8 Hz, *o*–*C*H); 126.5 (dp, ^3^*J*(C,P) = 10.0 Hz, ^3^*J*(C,F_eq_) = 4.9 Hz, *m*–*C*H) ppm. The resonance for the CO ligand was not observed. IR (ATR): 1986 (C≡O), 1940 (C≡O), 824 (S–F) cm^−1^.

When the reaction was performed with ^13^CO, the complex **8′** was obtained.

Selected NMR data: ^31^P{^1^H} NMR (121.5 MHz, CD_2_Cl_2_): δ = 0.84 (t, ^2^*J*(P,C) = 13.5 Hz) ppm. ^13^C{^1^H} NMR (75.4 MHz, CD_2_Cl_2_): 179.6 (t, ^2^*J*(C,P) = 13.6 Hz, *^13^C*O) ppm. IR (ATR): 1933 (C≡O), 1896 (C≡O), 824 (S–F) cm^−1^.

### 3.10. Formation of Trans,trans-[RhCl(CO)_2_{P(p-C_6_H_4_SF_5_)_3_}_2_] (9)

Complex [RhCl(COD){P(*p*-C_6_H_4_SF_5_)_3_}] (**5**) (20 mg, 0.02 mmol) was dissolved in C_6_D_6_ (0.4 mL) and the phosphine **1** (14 mg, 0.02 mmol) was added. Then, the solution was cooled to 77 K, degassed and treated with CO. After 10 min, the solution turned clear and a yellow precipitate was formed. The solid was filtered off and washed with C_6_D_6_ (2 × 0.25 mL) and dried under vacuum Yield: 29 mg (89%).

^31^P{^1^H} NMR (121.5 MHz, acetone-*d_6_*): δ = 27.6 (s, br) ppm. ^31^P{^1^H} NMR (121.5 MHz, 243 K, acetone-*d_6_*): δ = 29.15 (d, br, ^2^*J*(P,Rh) ≈ 130 Hz) ppm. ^1^H NMR (300.1 MHz, acetone-*d^6^*): δ 8.22–8.00 (m, *m*–C*H* + *o*–C*H*) ppm. ^19^F NMR (282.4 MHz, 243 K acetone-*d^6^*): δ = 82.9 (p, ^2^*J*(F,F) = 148 Hz, 1F, S*F*_5_); 61.6 (d, ^2^*J*(F,F) = 148 Hz, 4F, S*F*_5_) ppm. ^13^C{^1^H} NMR (75.4 MHz, acetone-*d^6^*): 156.1–154.7 (m, *C_q_*–SF_5_); 136.3 (s br, P–*C*_q_ + *o*–*C*H); 127.0 (s br, *m*–*C*H) ppm. The resonance for the CO ligand was not observed. IR (ATR): 1992 (C≡O), 828 (S–F) cm^−1^.

When the reaction was performed with ^13^CO, complex **9′** was obtained.

Selected NMR data: ^31^P{^1^H} NMR (121.5 MHz, acetone-*d_6_*): δ = 28.62 (s, br) ppm. ^31^P{^1^H} NMR (121.5 MHz, 203 K, acetone-*d^6^*): δ = 29.49 (d, br, ^2^*J*(P,Rh) ≈ 130 Hz) ppm. ^13^C{^1^H} NMR (75.4 MHz, acetone-*d^6^*): 187.4 (t, ^1^*J*(C,Rh) = 73.0 Hz, *^13^C*O) ppm. ^13^C{^1^H} NMR (75.4 MHz, 203 K, acetone-*d^6^*): 187.1 (d, ^1^*J*(C,Rh) = 72.6 Hz, *^13^C*O) ppm. IR (ATR): 1945 (C≡O), 824 (S–F) cm^−1^.

### 3.11. X-ray Diffraction Analysis

For the structure determination, the data collection was performed with a BRUKER APEX-II CCD diffractometer (Bruker AXS, Karlsruhe, Germany) using Mo-K_α_ radiation (λ = 0.71073 Å). Multi-scan absorption corrections implemented in SADABS [71] were applied to the data. The structures were solved by intrinsic phasing method (SHELXT-2014) [72] and refined by full–matrix least square procedures based on F2 with all measured reflections (SHELXL-2018) [73], with anisotropic temperature factors for all non-hydrogen atoms. In complex **4·0.5C_6_H_6_**, C(sp^3^) atoms of COD ligand were treated with ISOR while in complex **6·C_6_H_6_**, all the carbon atoms of the solvent molecule were treated with EADP. All carbon bound hydrogen atoms were added geometrically and refined by using a riding model. CCDC 2021751–2021754 contain the supplementary crystallographic data. These data can be obtained free of charge via http://www.ccdc.cam.ac.uk/conts/retrieving.html (or from the CCDC, 12 Union Road, Cambridge CB2 1EZ, UK; Fax: +44 1223 336033; E–mail: deposit@ccdc.cam.ac.uk).

*Crystal Data for***1·C_6_H_6_**. C_18_H_12_F_15_PS_3_·C_6_H_6_, *M*_W_ 718.53, triclinic, space group *P*$\bar{1}$, *a*: 10.0772(6) Å, *b*: 10.2132(6) Å, *c*: 15.1197(12) Å, *α*: 70.446(3)°, *β*: 70.770(4)°, *γ* = 85.849(3)°, *V* = 1383.31(16) Å^3^, *Z* = 2, *D*_calc_: 1.725 mg m^−3^, *T* = 103(2) K, *μ =* 0.443 mm^−1^. 28,056 measured reflections (2θ: 2.142–26.416°), 5658 unique (*R*_int_ = 0.0533). Final agreement factors were *R*^1^ = 0.0402 (*I* > 2σ(I)) and *wR*^2^ = 0.0862.

*Crystal Data for***2·2C_6_H_6_**. C_30_H_24_F_15_OPS_3_·2C_6_H_6_, *M*_W_ 812.64, triclinic, space group *P*$\bar{1}$, *a*: 7.7689(7) Å, *b*: 12.3481(10) Å, *c*: 17.6929(17) Å, *α*: 106.915(3)°, *β*: 90.217(4)°, *γ* = 92.514(3)°, *V* = 1622.1(3) Å^3^, *Z* = 2, *D*_calc_: 1.664 mg m^−3^, *T* = 102(2) K, *μ =* 0.391 mm^−1^. 65,957 measured reflections (2θ: 2.374–26.846°), 6935 unique (*R*_int_ = 0.0577). Final agreement factors were *R*^1^ = 0.0388 (*I* > 2σ(I)) and *wR*^2^ = 0.0890.

*Crystal Data for***4·0.5C_6_H_6_**. C_26_H_24_ClF_15_IrPS_3_·0.5C_6_H_6_, *M*_W_ 1015.30, monoclinic, space group *P2_1_/c*, *a*: 16.8589(11) Å, *b*: 17.1921(11) Å, *c*: 11.8495(8) Å, *β*: 101.744(2)°, *V* = 3362.6(4) Å^3^, *Z* = 2, *D*_calc_: 2.006 mg m^−3^, *T* = 100(2) K, *μ =* 4.390 mm^−1^. 41,026 measured reflections (2θ: 2.264–26.436°), 6901 unique (*R*_int_ = 0.0505). Final agreement factors were *R*^1^ = 0.0340 (*I* > 2σ(I)) and *wR*^2^ = 0.0763.

*Crystal Data for***6·C_6_H_6_**. C_52_H_52_Cl_2_F_30_Rh_2_P_2_S_6_·C_6_H_6_, *M*_W_ 1856.06, monoclinic, space group *C2/c*, *a*: 19.8800(13) Å, *b*: 11.6762(7) Å, *c*: 29.2074(19) Å, *β*: 96.426(2)°, *V* = 6737.1(7) Å^3^, *Z* = 4, *D*_calc_: 1.830 mg m^−3^, *T* = 103(2) K, *μ =* 0.926 mm^−1^. 62,530 measured reflections (2θ: 2.182–24.840°), 5816 unique (*R*_int_ = 0.1005). Final agreement factors were *R*^1^ = 0.0431 (*I* > 2σ(I)) and *wR*^2^ = 0.0910.

### 3.12. Computational Details

Calculations were run using the Gaussian 09 (Revision D.01, Gaussian, Inc., Wallingford, CT, USA) program package [74]. In the case of phosphines and phosphine radical cations the CAM-B3LYP functional was used and 6-311G(d,p) basis set were employed for all atoms. For the nickel complexes, the B3LYP functional was chosen. Nickel was described with RECPs and the associated LANL2DZ basis sets [75] while the ligands were described with 6-31G(d,p). All calculated structures were identified as minima (no negative eigenvalues). All xyz coordinates are included as Supplementary Material (Tables S1–S3).

## 4. Conclusions

An unprecedented phosphine bearing SF_5_ groups, P(*p*-C_6_H_4_SF_5_)_3_ (**1**), was successfully synthesized. It exhibits a moderate air stability, which is corroborated experimentally and by theoretical methods. The HOMO energy level and the phosphorus-selenium coupling constant of the synthesized phosphine selenide indicate that **1** is a weaker donor than P(*p*-C_6_H_4_CF_3_)_3_ and very close to P(*m*-C_6_H_4_(CF_3_)_2_)_3_. The calculated Tolman electronic parameter are consistent with an electron-withdrawing character. The molecular structures of iridium and rhodium complexes allowed for an estimation of the cone angle, which is slightly larger than the one for P(*p*-C_6_H_4_CF_3_)_3_. Finally, reactivity studies of [MCl(COD){P(*p*-C_6_H_4_SF_5_)_3_}] (M = Ir (**4**), Rh (**5**)) towards CO revealed a preference for the formation of the diphosphine complexes [MCl(CO)_2_{P(*p*-C_6_H_4_SF_5_)_3_}_2_] (M = Ir (**8**), Rh (**9**)) instead of [MCl(CO)_2_{P(*p*-C_6_H_4_SF_5_)_3_}]. Overall, the presence of SF_5_ groups in the phosphine **1** qualifies it as an electron-poor and sterically demanding phosphine. This might provide new opportunities for applications in catalysis.

## Figures and Tables

**Table 1:** DFT calculated SOMO energies (eV) of triarylphosphines radical cations [PAr_3_]^•+^ (CAM-B3LYP/6-311G(d,p)).

Ar Group	SOMO	Ar Group	SOMO
*p*-C_6_H_4_SF_5_	−12.22	3,4,5-C_6_H_2_F_3_	–11.93
*p*-C_6_H_4_CF_3_	−11.82	*p*-C_6_H_4_Me	–10.62
*m*-C_6_H_3_(CF_3_)_2_	–12.36	*p*-C_6_H_4_OMe	–10.01
*p*-C_6_H_4_F	–11.21	C_6_H_5_	−11.11

**Table 2:** Calculated HOMO energies and TEP values of triarylphosphines (PAr_3_) and experimental ^77^Se NMR data of the corresponding selenides.

Ar Group	HOMO (eV)	TEP (cm^−1^) [a]	^1^*J*_Se-P_ (Hz)
*p*-C_6_H_4_SF_5_	−8.69	2072.6	792
*p*-C_6_H_4_CF_3_	−8.17	2069.3	765 [b]
*m*-C_6_H_3_(CF_3_)_2_	−8.78	2075.2	802 [b]
*p*-C_6_H_4_F	−7.58	*n.d.*	741 [b]
3,4,5-C_6_H_2_F_3_	−8.36	*n.d.*	792 [c]
*p*-C_6_H_4_Me	−7.10	2061.7 (2166.7)	715 [b]
*p*-C_6_H_4_OMe	−6.78	*n.d.*	710 [b]
C_6_H_5_	−7.34	2063.5 (2068.9)	733 [b]

*n.d*. = not determined; [a] Calculated values from calculated ν_CO_ (A_1_ band) of Ni(CO)_3_L complexes using the relation TEP = 0.9572ν_CO_ + 4.081 [54]. In brackets experimentally determined values given in ref. [52] [b] Values given in ref. [50]; [c] Value given in ref. [55].

## Supplementary Materials

The Supplementary Materials are available online.

## References

[1] Crabtree R.H. (2014). The Organometallic Chemistry of the Transition Metals.

[2] Hartwig J.F. (2010). Organotransition Metal Chemistry: From Bonding to Catalysis.

[3] McAuliffe C.A., Levason W. (1999). Phosphine, Arsine and Stibine Complexes of the Transition Elements.

[4] Pignolet L.H. (1983). Homogeneous Catalysis with Metal Phosphine Complexes.

[5] Pollock C.L., Saunders G.C., Smyth E.C.M.S., Sorokin V.I. (2008). Fluoroarylphosphines as ligands. J. Fluor. Chem..

[6] Clarke M.L., Ellis D., Mason K.L., Orpen A.G., Pringle P.G., Wingad R.L., Zaher D.A., Baker R.T. (2005). The electron-poor phosphines P{C_6_H_3_(CF_3_)_2_-3,5}_3_ and P(C_6_F_5_)_3_ do not mimic phosphites as ligands for hydroformylation. A comparison of the coordination chemistry of P{C_6_H_3_(CF_3_)_2_-3,5}_3_ and P(C_6_F_5_)_3_ and the unexpectedly low hydroformylation activity of their rhodium complexes. Dalton Trans..

[7] Fawcett J., Hope E.G., Kemmitt R.D., Paige D.R., Russell D.R., Stuart A.M., Stuart M. (1998). Platinum group metal complexes of arylphosphine ligands containing perfluoroalkyl ponytails; crystal structures of [RhCl_2_(*η*^5^-C_5_Me_5_){P(C_6_H_4_C_6_F_13_-4)_3_}] and cis- and trans-[PtCl_2_{P(C_6_H_4_C_6_F_13_-4)_3_}_2_]. J. Chem. Soc. Dalton Trans..

[8] Hope E.G., Kemmitt R.D.W., Paige D.R., Stuart A.M., Wood D.R.W. (1999). Synthesis and coordination chemistry of meta-perfluoroalkyl-derivatised triarylphosphines. Polyhedron.

[9] Corcoran C., Fawcett J., Friedrichs S., Holloway J.H., Hope E.G., Russell D.R., Saunders G.C., Stuart A.M. (2000). Structural and electronic impact of fluorine in the ortho positions of triphenylphosphine and 1,2-bis(diphenylphosphino)ethane; a comparison of 2,6-difluorophenyl- with pentafluorophenyl-phosphines. J. Chem. Soc. Dalton Trans..

[10] Croxtall B., Fawcett J., Hope E.G., Stuart A.M. (2002). Synthesis and coordination chemistry of ortho-perfluoroalkyl-derivatised triarylphosphines. J. Chem. Soc. Dalton Trans..

[11] Saunders G.C. (2015). Structural and electronic properties of tris(4-trifluoromethyltetrafluorophenyl)phosphine. J. Fluor. Chem..

[12] Uson R., Oro L.A., Fernandez M.J. (1980). Preparation, reactions and catalytic activity of complexes of the type [Ir(COD){P(*p*-RC_6_H_4_)_3_}_2_]A (R = Cl, F, H, CH_3_ or CH_3_O.; A = ClO_4_^−^ or B(C_6_H_5_)_4_^−^). J. Organomet. Chem..

[13] Matsubara K., Fujii T., Hosokawa R., Inatomi T., Yamada Y., Koga Y. (2019). Fluorine-Substituted Arylphosphine for an NHC-Ni(I) System, Air-Stable in a Solid State but Catalytically Active in Solution. Molecules.

[14] Moser W.R., Papile C.J., Brannon D.A., Duwell R.A., Weininger S.J. (1987). The mechanism of phosphine-modified rhodium-catalyzed hydroformylation studied by CIR-FTIR. J. Mol. Catal..

[15] Chen C., Wang Y., Shi X., Sun W., Zhao J., Zhu Y.-P., Liu L., Zhu B. (2020). Palladium-Catalyzed C-2 and C-3 Dual C–H Functionalization of Indoles: Synthesis of Fluorinated Isocryptolepine Analogues. Org. Lett..

[16] Paterson A.J., Dunås P., Rahm M., Norrby P.-O., Kociok-Köhn G., Lewis S.E., Kann N. (2020). Palladium Catalyzed Stereoselective Arylation of Biocatalytically Derived Cyclic 1,3-Dienes: Chirality Transfer via a Heck-Type Mechanism. Org. Lett..

[17] Jakab A., Dalicsek Z., Holczbauer T., Hamza A., Pápai I., Finta Z., Timári G., Soós T. (2015). Superstable Palladium(0) Complex as an Air- and Thermostable Catalyst for Suzuki Coupling Reactions. Eur. J. Org. Chem..

[18] Cheng L., Li M.-M., Wang B., Xiao L.-J., Xie J.-H., Zhou Q.-L. (2019). Nickel-catalyzed hydroalkylation and hydroalkenylation of 1,3-dienes with hydrazones. Chem. Sci..

[19] Sheppard W.A. (1962). The Electrical Effect of the Sulfur Pentafluoride Group. J. Am. Chem. Soc..

[20] Hansch C., Muir R.M., Fujita T., Maloney P.P., Geiger F., Streich M. (1963). The Correlation of Biological Activity of Plant Growth Regulators and Chloromycetin Derivatives with Hammett Constants and Partition Coefficients. J. Am. Chem. Soc..

[21] Hansch C., Leo A., Taft R.W. (1991). A survey of Hammett substituent constants and resonance and field parameters. Chem. Rev..

[22] Savoie P.R., Welch J.T. (2015). Preparation and Utility of Organic Pentafluorosulfanyl-Containing Compounds. Chem. Rev..

[23] Lentz D., Seppelt K., Akiba K.-Y. (1999). The -SF_5_, -SeF_5_, and -TeF_5_ Groups in Organic Chemistry. Chemistry of Hypervalent Compounds.

[24] Gard G.L. (2009). Recent Milestones in SF_5_-Chemistry. Chim. Oggi.

[25] Altomonte S., Zanda M. (2012). Synthetic chemistry and biological activity of pentafluorosulphanyl (SF_5_) organic molecules. J. Fluor. Chem..

[26] Kanishchev O.S., Dolbier W.R., Scriven E.F.V., Ramsden C.A. (2016). Chapter One—SF_5_-Substituted Aromatic Heterocycles. Advances in Heterocyclic Chemistry.

[27] Chan J.M.W. (2019). Pentafluorosulfanyl group: An emerging tool in optoelectronic materials. J. Mater. Chem. C.

[28] Beier P. (2017). Synthesis and reactivity of novel sulfur pentafluorides—Effect of the SF_5_ group on reactivity of nitrobenzenes in nucleophilic substitution. Phosphorus Sulfur Silicon Relat. Elem..

[29] Beier P., Ma J.-A., Cahard D. (2020). Pentafluorosulfanylation of Aromatics and Heteroaromatics. Emerging Fluorinated Motifs.

[30] Haufe G., Ma J.-A., Cahard D. (2020). Pentafluorosulfanylation of Aliphatic Substrates. Emerging Fluorinated Motifs.

[31] Damerius R., Leopold D., Schulze W., Seppelt K. (1989). Strukturen von SF_5_-substituierten Metallkomplexen. Z. Anorg. Allg. Chem..

[32] Henkel T., Klauck A., Seppelt K. (1995). Pentafluoro-λ^6^-sulfanylacetylene complexes of cobalt. J. Organomet. Chem..

[33] Preugschat D., Thrasher J.S. (1996). Pentacarbonylchrom-Komplexe SF_5_-substituierter Isocyanide. Z. Anorg. Allg. Chem..

[34] Shavaleev N.M., Xie G., Varghese S., Cordes D.B., Slawin A.M.Z., Momblona C., Ortí E., Bolink H.J., Samuel I.D.W., Zysman-Colman E. (2015). Green Phosphorescence and Electroluminescence of Sulfur Pentafluoride-Functionalized Cationic Iridium(III) Complexes. Inorg. Chem..

[35] Ma X.-F., Luo X.-F., Yan Z.-P., Wu Z.-G., Zhao Y., Zheng Y.-X., Zuo J.-L. (2019). Syntheses, Crystal Structures, and Photoluminescence of a Series of Iridium(III) Complexes Containing the Pentafluorosulfanyl Group. Organometallics.

[36] Pal A.K., Henwood A.F., Cordes D.B., Slawin A.M.Z., Samuel I.D.W., Zysman-Colman E. (2017). Blue-to-Green Emitting Neutral Ir(III) Complexes Bearing Pentafluorosulfanyl Groups: A Combined Experimental and Theoretical Study. Inorg. Chem..

[37] Groves L.M., Schotten C., Beames J., Platts J.A., Coles S.J., Horton P.N., Browne D.L., Pope S.J.A. (2017). From Ligand to Phosphor: Rapid, Machine-Assisted Synthesis of Substituted Iridium(III) Pyrazolate Complexes with Tuneable Luminescence. Chem. A Eur. J..

[38] Henwood A.F., Webster J., Cordes D., Slawin A.M.Z., Jacquemin D., Zysman-Colman E. (2017). Phosphorescent platinum(ii) complexes bearing pentafluorosulfanyl substituted cyclometalating ligands. RSC Adv..

[39] Berg C., Braun T., Laubenstein R., Braun B. (2016). Palladium-mediated borylation of pentafluorosulfanyl functionalized compounds: The crucial role of metal fluorido complexes. Chem. Commun..

[40] Golf H.R.A., Reissig H.-U., Wiehe A. (2015). Synthesis of SF_5_-Substituted Tetrapyrroles, Metalloporphyrins, BODIPYs, and Their Dipyrrane Precursors. J. Org. Chem..

[41] Berry A.D., De Marco R.A. (1982). Reaction of pentafluoro[(trifluoromethyl)acetylenyl]sulfur with nickel tetracarbonyl. Inorg. Chem..

[42] Sergeeva T.A., Dolbier W.R. (2004). A New Synthesis of Pentafluorosulfanylbenzene. Org. Lett..

[43] Dunne B.J., Orpen A.G. (1991). Triphenylphosphine: A redetermination. Acta Cryst..

[44] Eapen K.C., Tamborski C. (1980). The synthesis of tris-(trifluoromethylphenyl)phosphines and phosphine oxides. J. Fluor. Chem..

[45] See R.F., Dutoi A.D., Fettinger J.C., Nicastro P.J., Ziller J.W. (1998). The crystal structures of (*p*-ClPh)_3_PO and (*p*-OMePh)_3_PO, including an analysis of the P-O bond in triarylphosphine oxides. J. Chem. Crystallogr..

[46] Stewart B., Harriman A., Higham L.J. (2011). Predicting the Air Stability of Phosphines. Organometallics.

[47] Dunne B.J., Morris R.B., Orpen A.G. (1991). Structural systematics. Part 3. Geometry deformations in triphenylphosphine fragments: A test of bonding theories in phosphine complexes. J. Chem. Soc. Dalton Trans..

[48] Palau C., Berchadsky Y., Chalier F., Finet J.-P., Gronchi G., Tordo P. (1995). Tris(monochlorophenyl)- and Tris(dichlorophenyl)phosphines: Molecular Geometry, Anodic Behavior, and ESR Studies. J. Phys. Chem..

[49] Chevykalova M.N., Manzhukova L.F., Artemova N.V., Luzikov Y.N., Nifant’ev I.E., Nifant’ev E.E. (2003). Electron-donating ability of triarylphosphines and related compounds studied by 31P NMR spectroscopy. Russ. Chem. Bull..

[50] Howell J.S., Lovatt J., Yates P., Gottlieb H., Hursthouse M., Light M. (1999). Effect of fluorine and trifluoromethyl substitution on the donor properties and stereodynamical behaviour of triarylphosphines. J. Chem. Soc. Dalton Trans..

[51] Allen D.W., Taylor B.F. (1982). The chemistry of heteroarylphosphorus compounds. Part 15. Phosphorus-31 nuclear magnetic resonance studies of the donor properties of heteroarylphosphines towards selenium and platinum(II). J. Chem. Soc. Dalton Trans..

[52] Tolman C.A. (1977). Steric effects of phosphorus ligands in organometallic chemistry and homogeneous catalysis. Chem. Rev..

[53] Gusev D.G. (2009). Donor Properties of a Series of Two-Electron Ligands. Organometallics.

[54] Perrin L., Clot E., Eisenstein O., Loch J., Crabtree R.H. (2001). Computed Ligand Electronic Parameters from Quantum Chemistry and Their Relation to Tolman Parameters, Lever Parameters, and Hammett Constants. Inorg. Chem..

[55] Kawaguchi S.-I., Minamida Y., Okuda T., Sato Y., Saeki T., Yoshimura A., Nomoto A., Ogawa A. (2015). Photoinduced Synthesis of P-Perfluoroalkylated Phosphines from Triarylphosphines and Their Application in the Copper-Free Cross-Coupling of Acid Chlorides and Terminal Alkynes. Adv. Synth. Catal..

[56] Ma X.-Y., Wang K., Zhang L., Li X.-J., Li R.-X. (2007). Selective Hydrogenation of Avermectin Catalyzed by Iridium-Phosphine Complexes. Chin. J. Chem..

[57] Tiburcio J., Bernès S., Torrens H. (2006). Electronic and steric effects of triarylphosphines on the synthesis, structure and spectroscopical properties of mononuclear rhodium(I)–chloride complexes. Polyhedron.

[58] Naaktgeboren A.J., Nolte R.J.M., Drenth W. (1980). Phosphorus-31 nuclear magnetic resonance studies of polymer-anchored rhodium(I) complexes. J. Am. Chem. Soc..

[59] Canepa G., Brandt C.D., Werner H. (2004). Mono- and Dinuclear Rhodium(I) and Rhodium(III) Complexes with the Bulky Phosphine 2,6-Me_2_C_6_H_3_CH_2_CH_2_P*t*Bu_2_, Including the First Structurally Characterized Cis-Configurated Dicarbonyl Compound, *cis*-[RhCl(CO)_2_(PR_3_)]. Organometallics.

[60] Müller T.E., Mingos D.M.P. (1995). Determination of the Tolman cone angle from crystallographic parameters and a statistical analysis using the crystallographic data base. Transit. Met. Chem..

[61] Reyna-Madrigal A., Ortiz-Pastrana N., Paz-Sandoval M.A. (2019). Cyclooctadiene iridium complexes with phosphine and pentadienyl ligands. J. Organomet. Chem..

[62] Bondi A. (1964). van der Waals Volumes and Radii. J. Phys. Chem..

[63] Joerg S., Drago R.S., Sales J. (1998). Reactivity of Phosphorus Donors. Organometallics.

[64] Ortega-Moreno L., Fernández-Espada M., Moreno J.J., Navarro-Gilabert C., Campos J., Conejero S., López-Serrano J., Maya C., Peloso R., Carmona E. (2016). Synthesis, properties, and some rhodium, iridium, and platinum complexes of a series of bulky m-terphenylphosphine ligands. Polyhedron.

[65] Von Hahmann C.N., Talavera M., Xu C., Braun T. (2018). Reactivity of 3,3,3-Trifluoropropyne at Rhodium Complexes: Development of Hydroboration Reactions. Chem. A Eur. J..

[66] Vaska L. (1966). Reversible Combination of Carbon Monoxide with a Synthetic Oxygen Carrier Complex. Science.

[67] Sanger A.R. (1985). Five-coordinate dicarbonyl complexes of rhodium(I): [RhX(CO)_2_(PPh_3_)_2_] (X = Cl, Br, I). Can. J. Chem..

[68] Perrin D.D., Armarego W.L.F. (1988). Purification of Laboratory Chemicals.

[69] Herde J.-L., Lambert J.C., Senoff C.V. (1974). Cyclooctene and 1,5-Cyclooctadiene Complexes of Iridium. Inorg. Synth..

[70] Van der Ent A., Onderdelinden A.L. (1973). Chlorobis(cyclooctene)rhodium(I) and -iridium(I) Complexes. Inorg. Synth..

[71] Sheldrick G.M. (1996). SADABS, Program for Empirical Absorption Correction of Area Detector Data, May 2014.

[72] Sheldrick G.M. (2013). SHELXT-2014, Program for the Solution of Crystal Structures from X-ray Data.

[73] Sheldrick G.M. (2018). SHELXL-2018, Program for the Refinement of Crystal Structures from X-ray Data.

[74] Frisch M.J., Schlegel H.B., Scuseria G.E., Robb M.A., Cheeseman J.R., Scalmani G., Barone V., Petersson G.A., Nakatsuji H., Li X. (2016). Gaussian 09, Revision D.01.

[75] Hay P.J., Wadt W.R. (1985). Ab initio effective core potentials for molecular calculations. Potentials for K to Au including the outermost core orbitals. J. Chem. Phys..

