# Supplementary material for: A SF5 Derivative of Triphenylphosphine as an Electron-Poor Ligand Precursor for Rh and Ir Complexes
Source: Molecules. 2020 Sep 1;25(17):3977. doi: 10.3390/molecules25173977 (PMC7504798; doi:10.3390/molecules25173977)
Supplement: Supplementary file 1 [file molecules-25-03977-s001.pdf]

Supporting Information

A SF_5 derivative of triphenylphosphine as an electron-poor ligand precursor for Rh and Ir complexes

Maria Talavera, Silke Hinze, Thomas Braun*, Reik Laubenstein and Roy Herrmann

Humboldt-Universität zu Berlin, Department of Chemistry, Brook-Taylor-Straße 2, D-12489 Berlin, Germany. E-mail: thomas.braun@chemie.hu-berlin.de

NMR Spectra

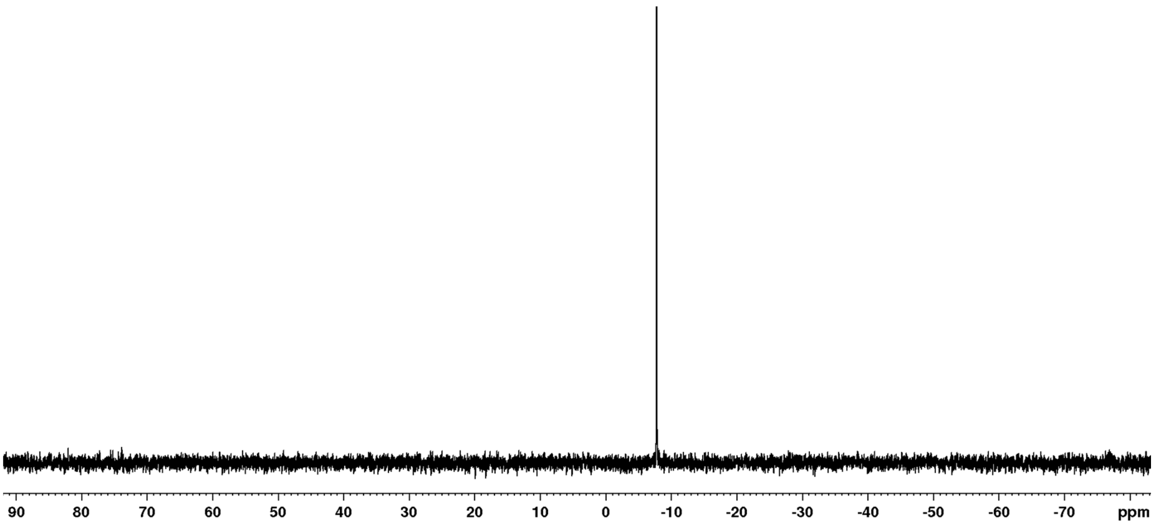

Figure S1. $^{31}P\{^1H\}$ NMR spectrum (121.5 MHz) of compound **1** in C_6D_6.

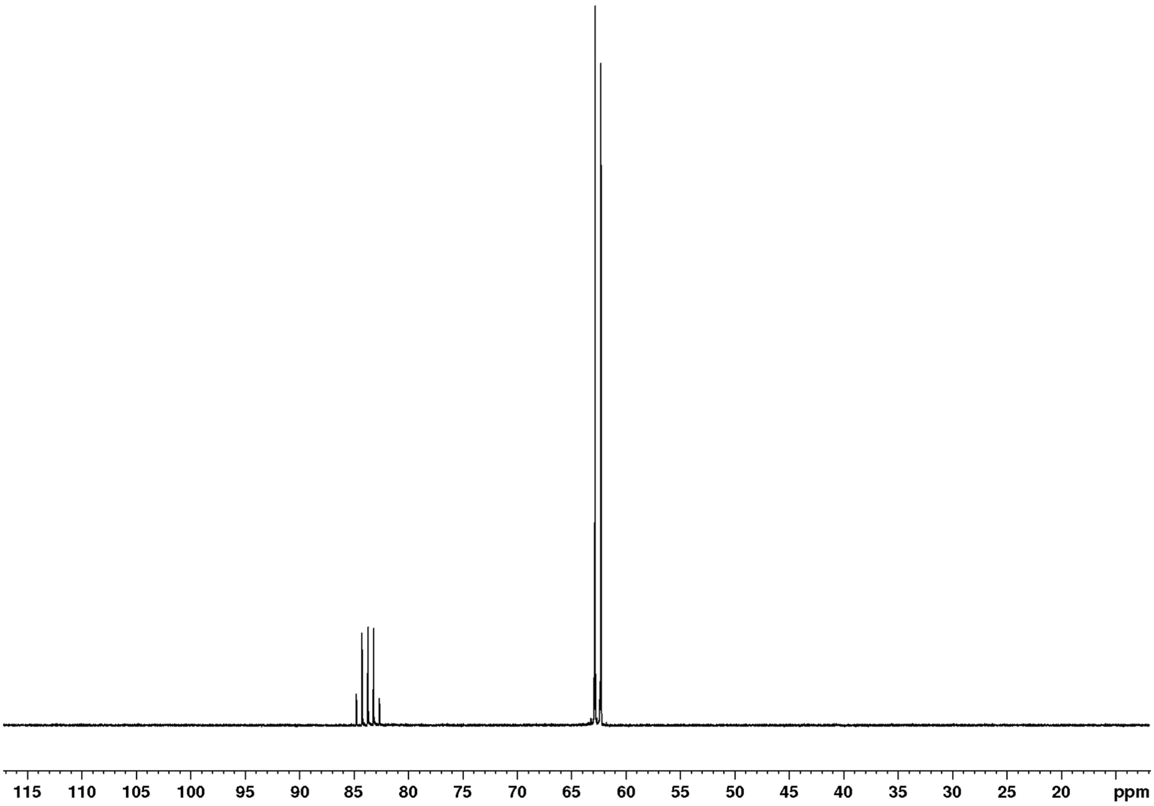

Figure S2. ^{19}F NMR spectrum (282.4 MHz) of compound **1** in C_6D_6.

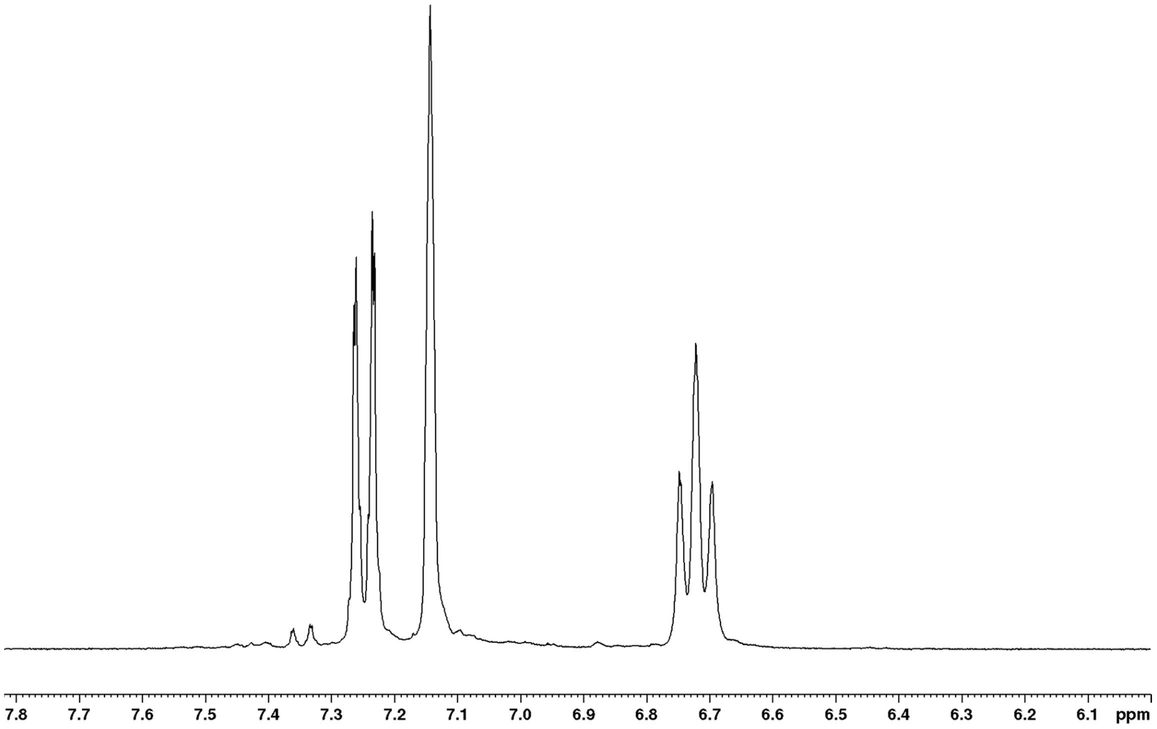

Figure S3. ^{1}H NMR spectrum (300.1 MHz) of compound **1** in C_6D_6.

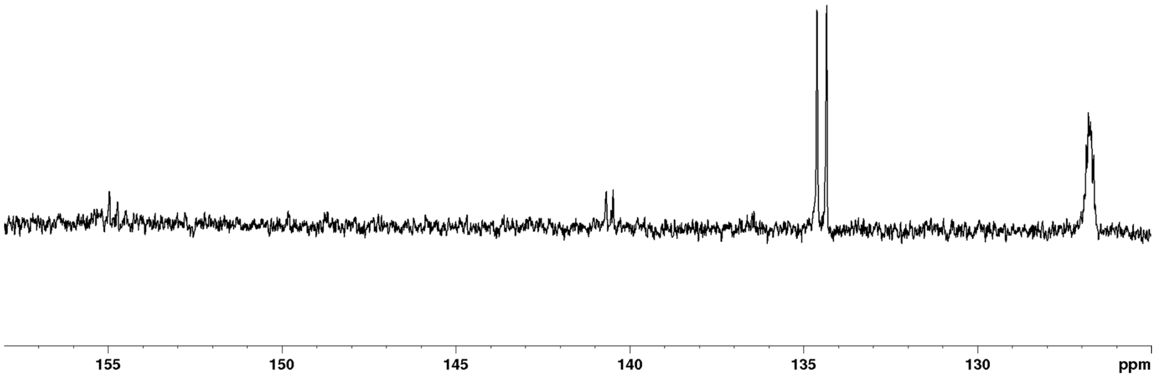

Figure S4. $^{13}C\{^{1}H\}$ NMR spectrum (75.4 MHz) of compound **1** in CD_2Cl_2.

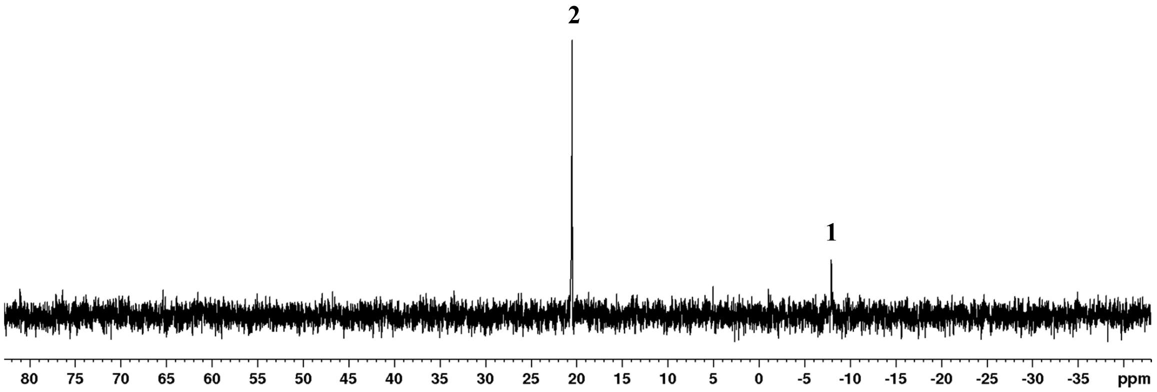

Figure S5. $^{31}P\{^{1}H\}$ NMR spectrum (121.5 MHz) of compound **2** in toluene-*d*^{8}.

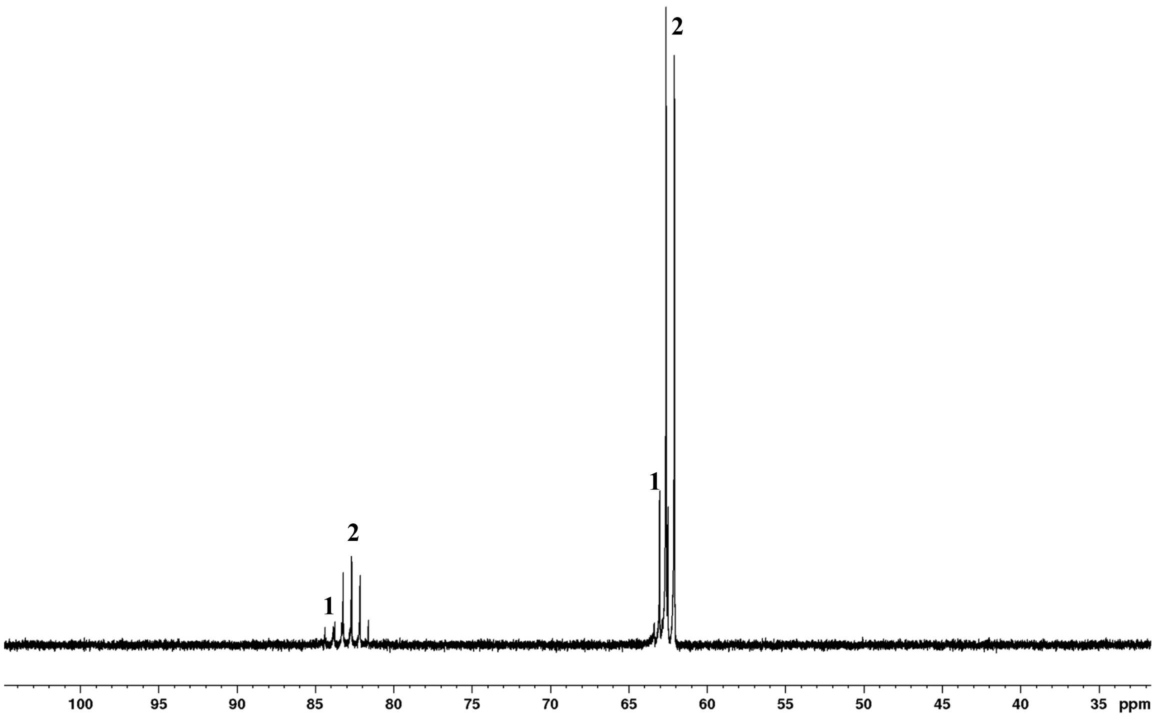

Figure S6. ^{19}F NMR spectrum (282.4 MHz) of compound **2** in toluene-*d*^{8}.

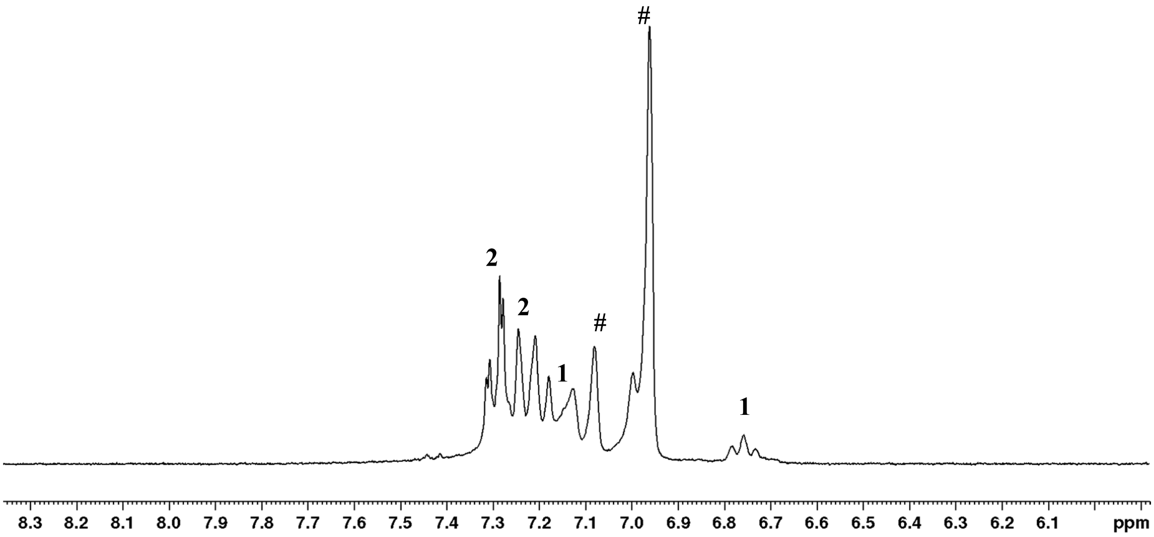

Figure S7. ^{1}H NMR spectrum (300.1 MHz) of compound **2** in toluene-d^8 (#).

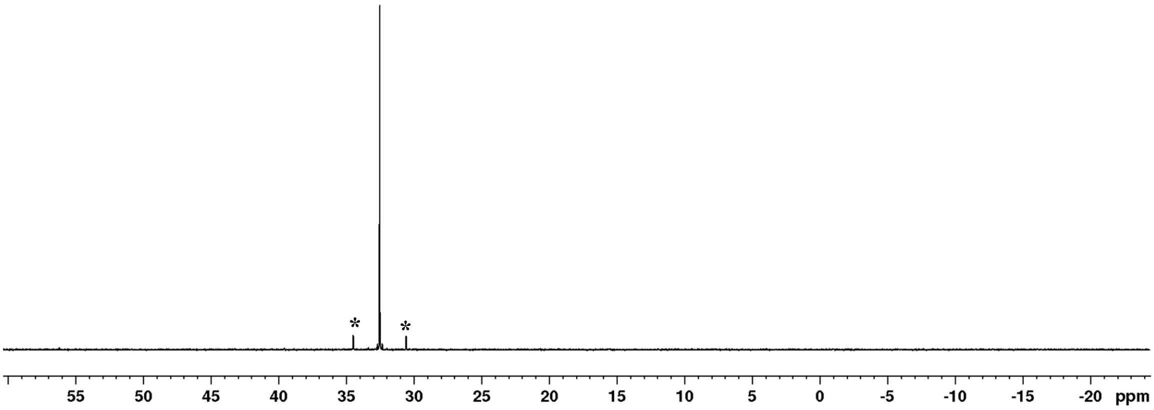

Figure S8. $^{31}P\{^{1}H\}$ NMR spectrum (202.4 MHz) of compound **3** in toluene-d^8. *^{77}Se satellites.

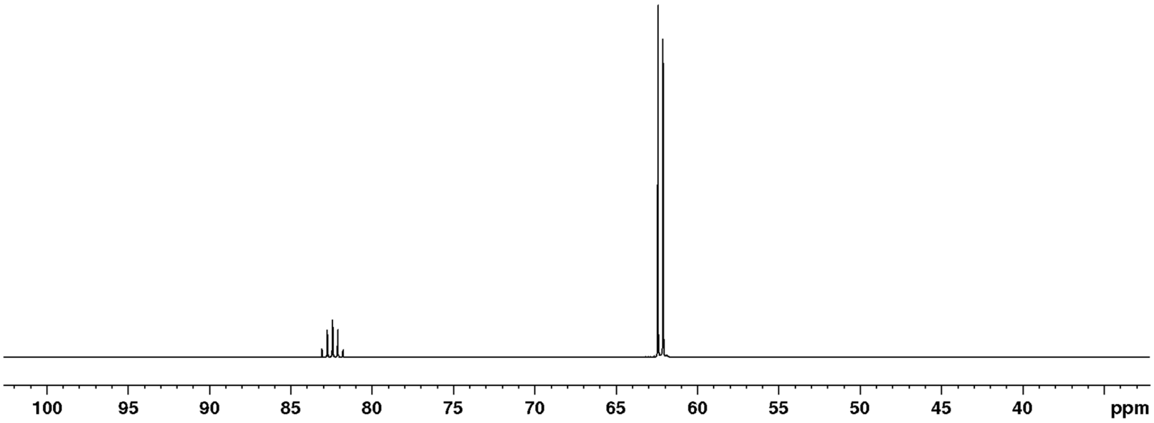

Figure S9. ^{19}F NMR spectrum (470.6 MHz) of compound **3** in toluene-d^8.

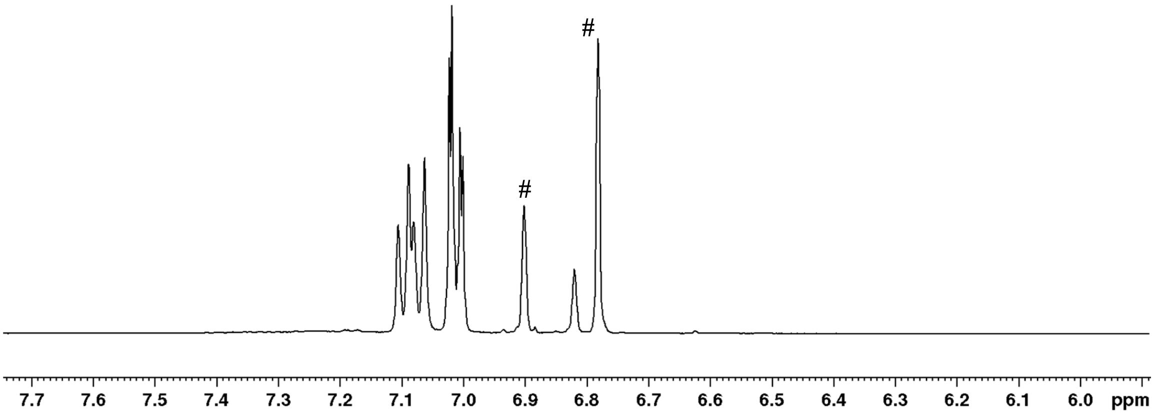

Figure S10. ^{1}H NMR spectrum (500.1 MHz) of compound **3** in toluene-d^8 (#).

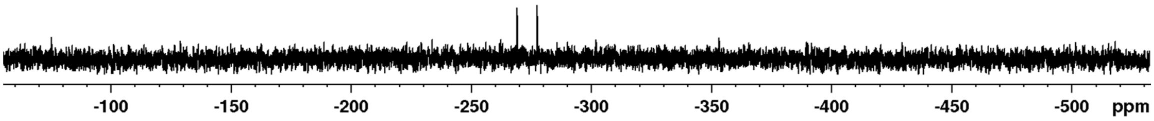

Figure S11. ^{77}Se NMR spectrum (95.4 MHz) of compound **3** in toluene-d^8.

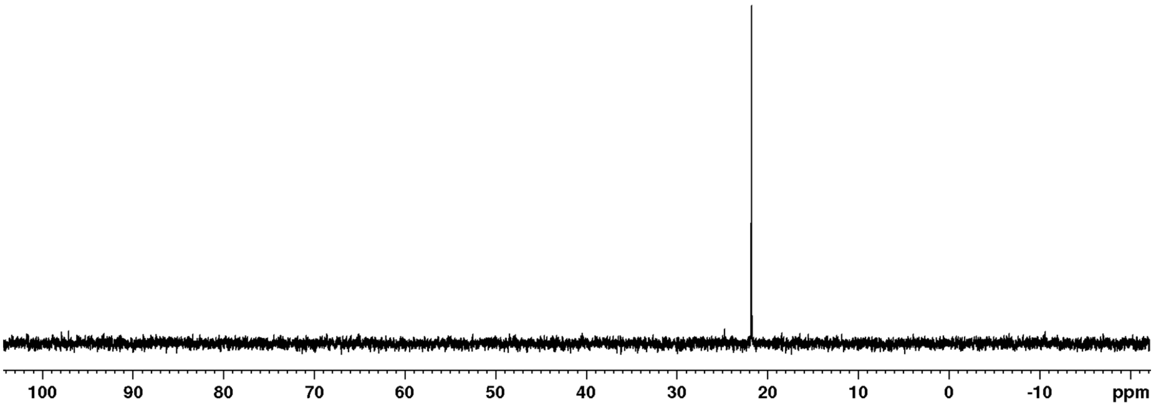

Figure S12. $^{31}P\{^{1}H\}$ NMR spectrum (121.5 MHz) of complex **4** in C_6D_6.

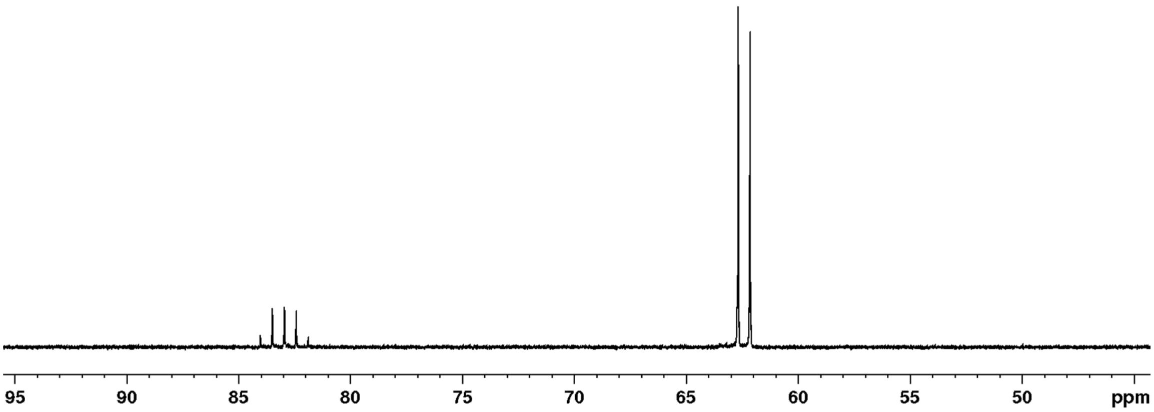

Figure S13. ^{19}F NMR spectrum (282.4 MHz) of complex **4** in C_6D_6.

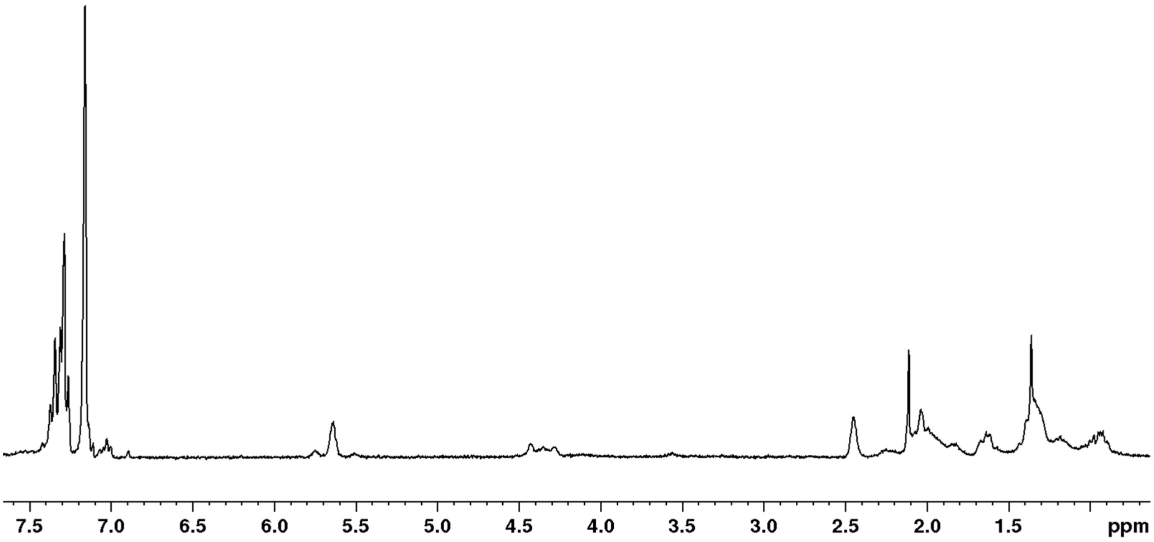

Figure S14. ^{1}H NMR spectrum (300.1 MHz) of complex **4** in C_6D_6.

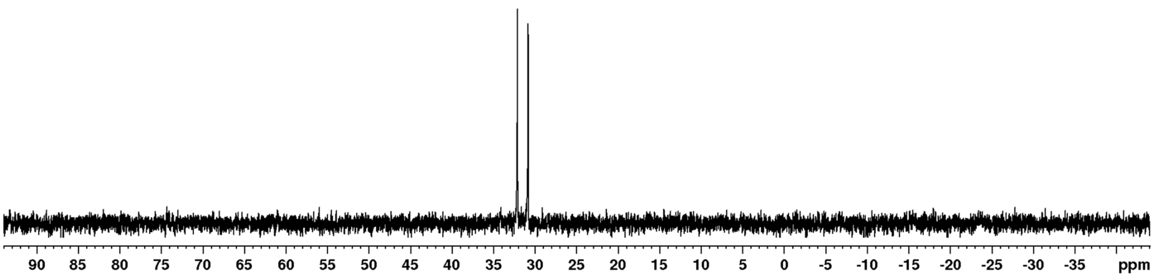

Figure S15. $^{31}P\{^{1}H\}$ NMR spectrum (121.5 MHz) of compound **5** in C_6D_6.

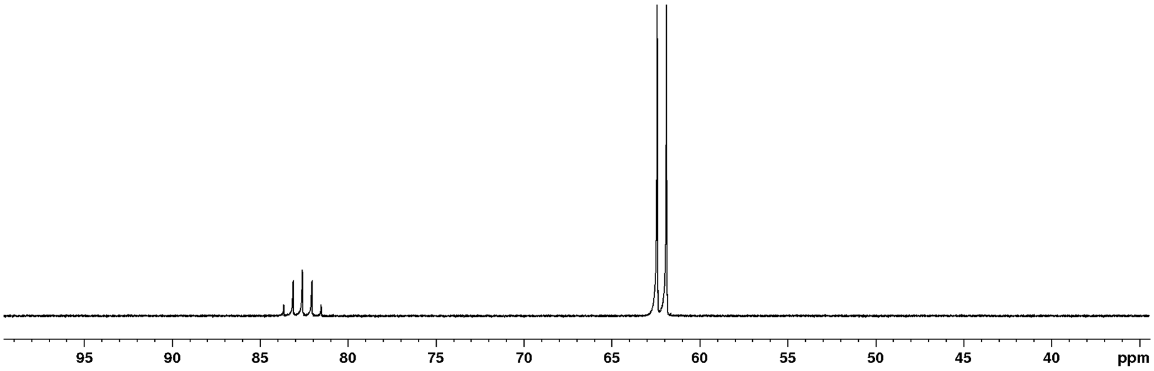

Figure S16. ^{19}F NMR spectrum (282.4 MHz) of compound **5** in C_6D_6.

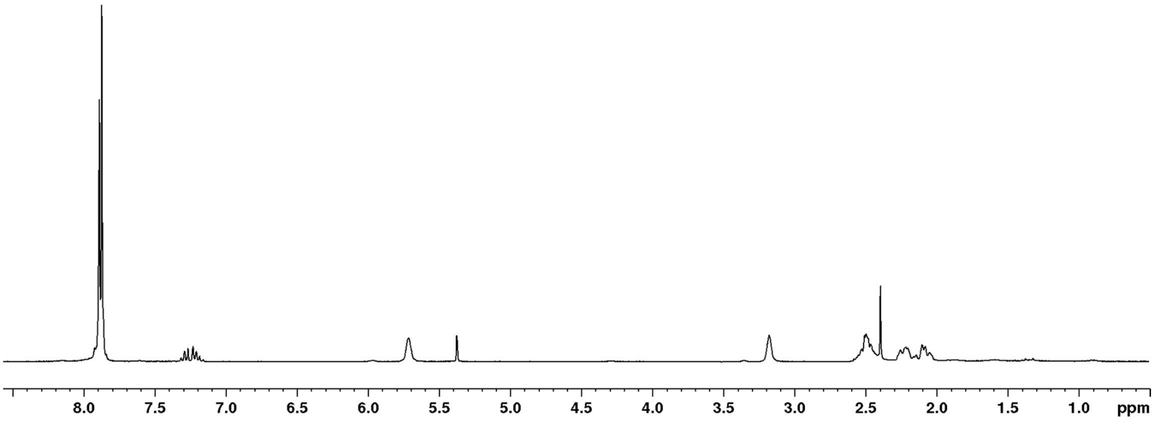

Figure S17. ^{1}H NMR spectrum (300.1 MHz) of compound **5** in C_6D_6.

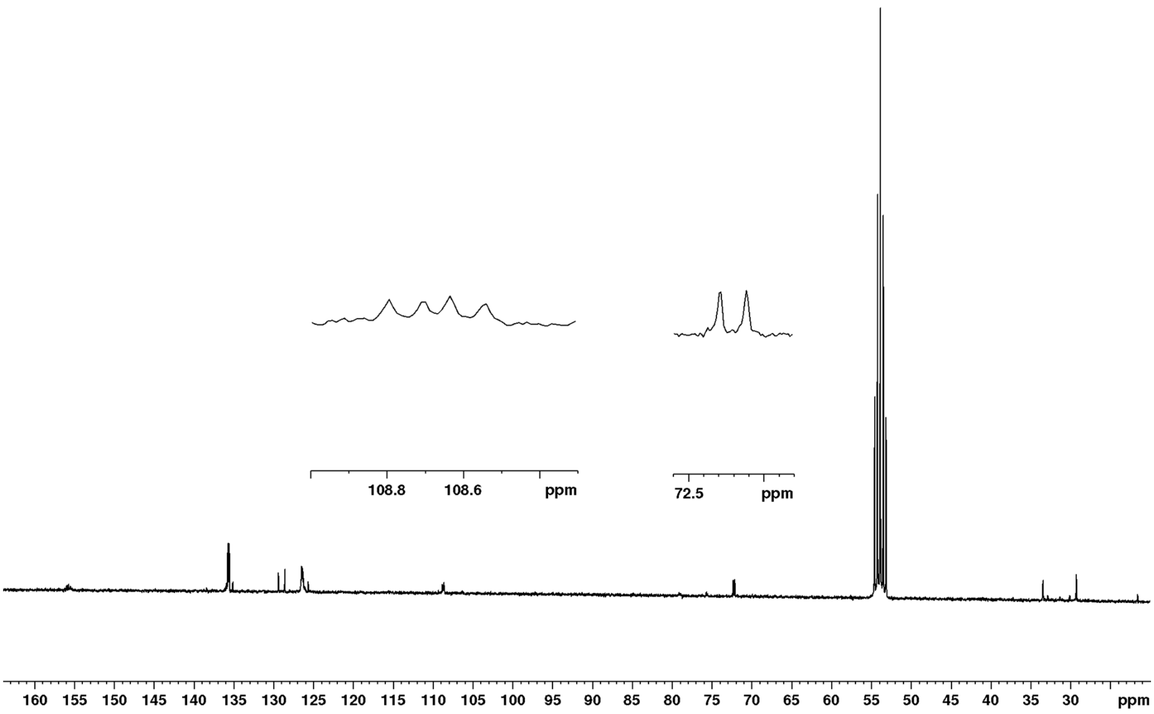

Figure S18. $^{13}C\{^1H\}$ NMR spectrum (75.4 MHz) of compound **6** in CD_2Cl_2.

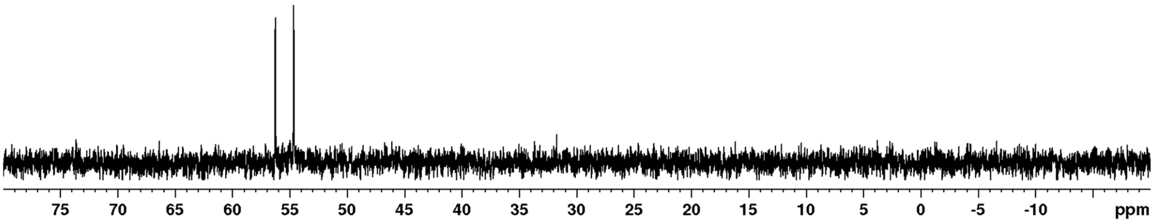

Figure S19. $^{31}P\{^1H\}$ NMR spectrum (121.5 MHz) of compound **6** in C_6D_6.

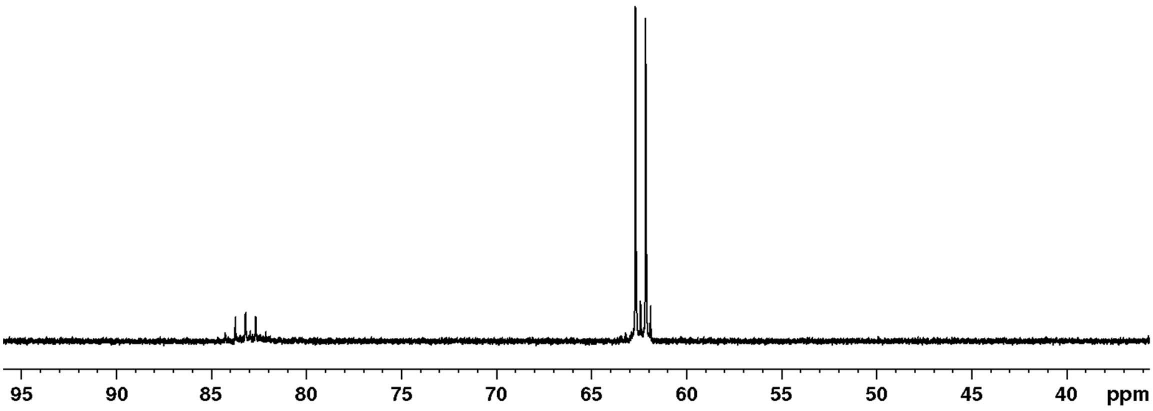

Figure S20. ^{19}F NMR spectrum (282.4 MHz) of compound **6** in C_6D_6.

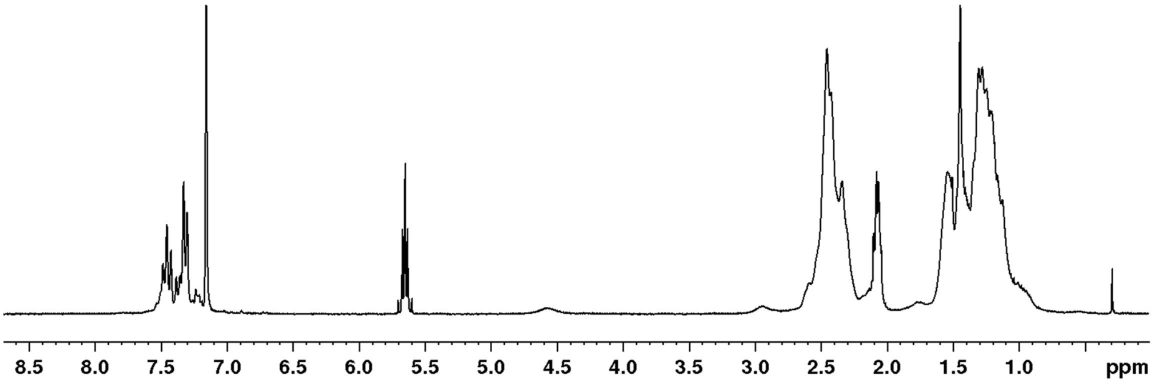

Figure S21. ^{1}H NMR spectrum (300.1 MHz) of compound **6** in C_6D_6.

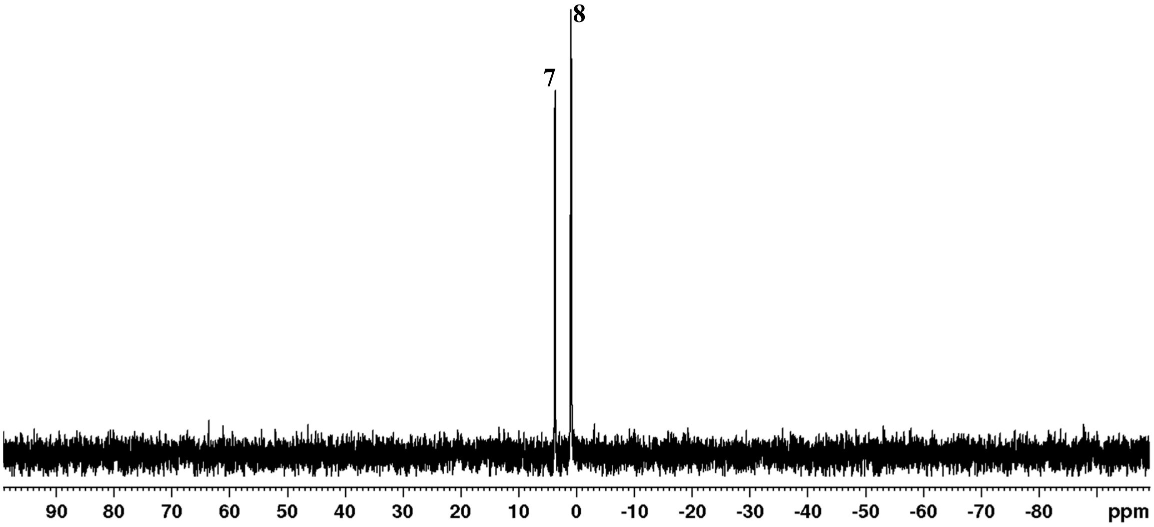

Figure S22. $^{31}P\{^{1}H\}$ NMR spectrum (121.5 MHz) of the mixture of complexes **7** and **8** in CD_2Cl_2.

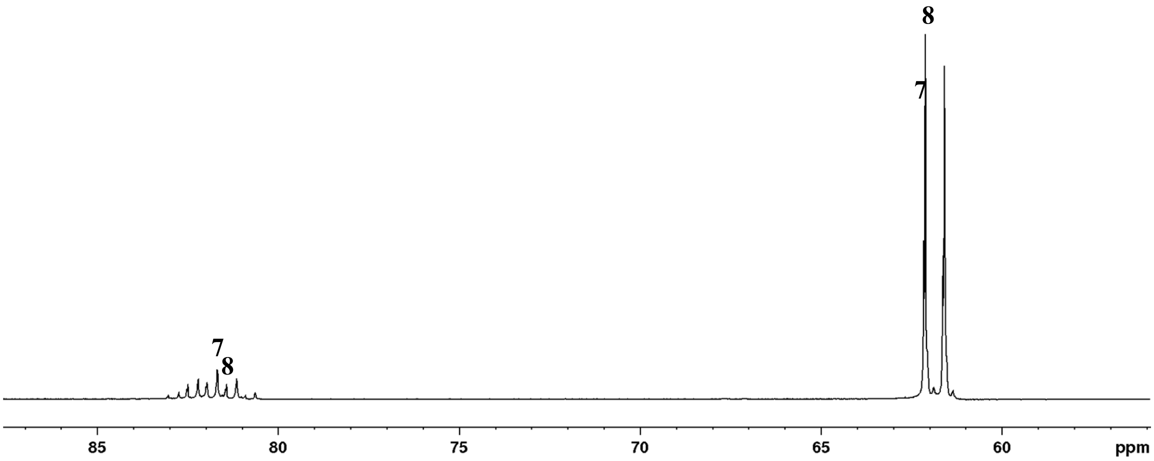

Figure S23. ^{19}F NMR spectrum (282.4 MHz) of the mixture of complexes **7** and **8** in CD_2Cl_2.

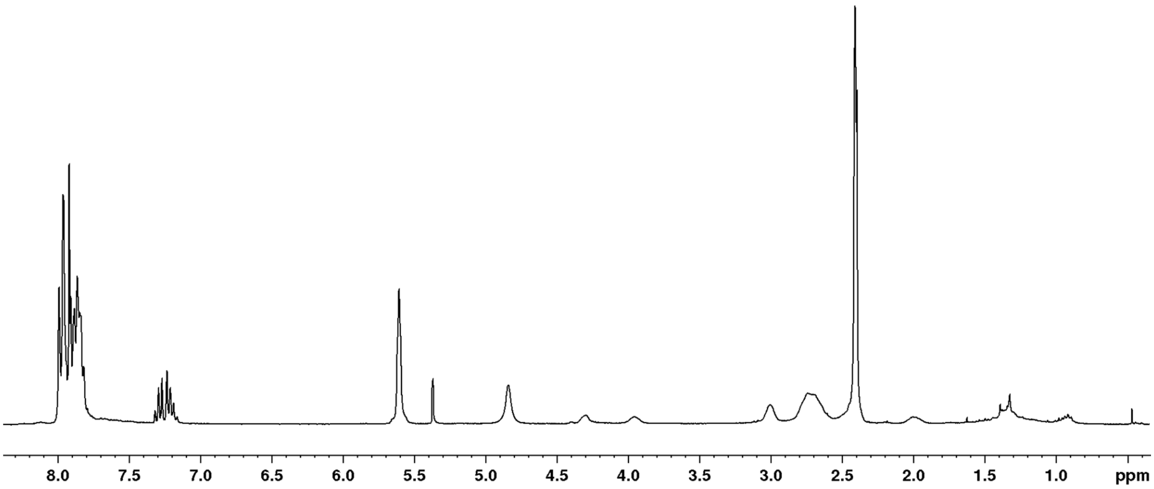

Figure S24. ^{1}H NMR spectrum (300.1 MHz) of the mixture of complexes **7** and **8** in CD_2Cl_2.

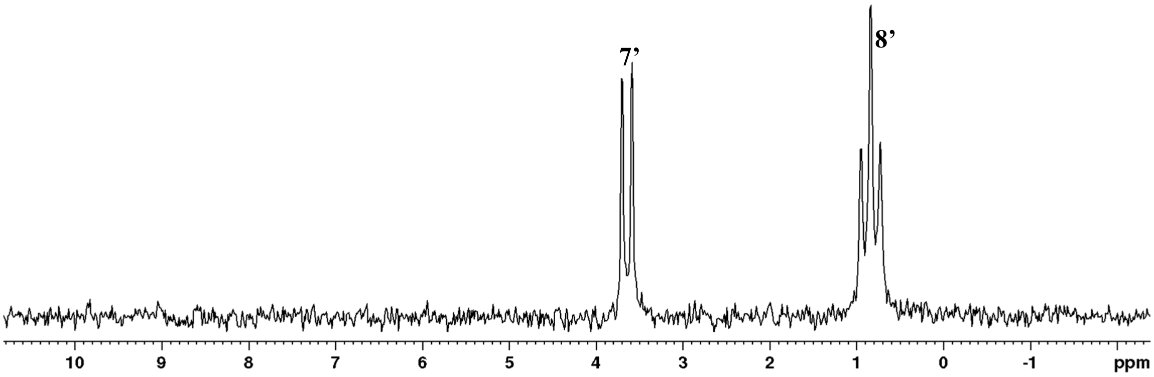

Figure S25. $^{31}P\{^{1}H\}$ NMR spectrum (121.5 MHz) of the mixture of complexes **7'** and **8'** in CD_2Cl_2.

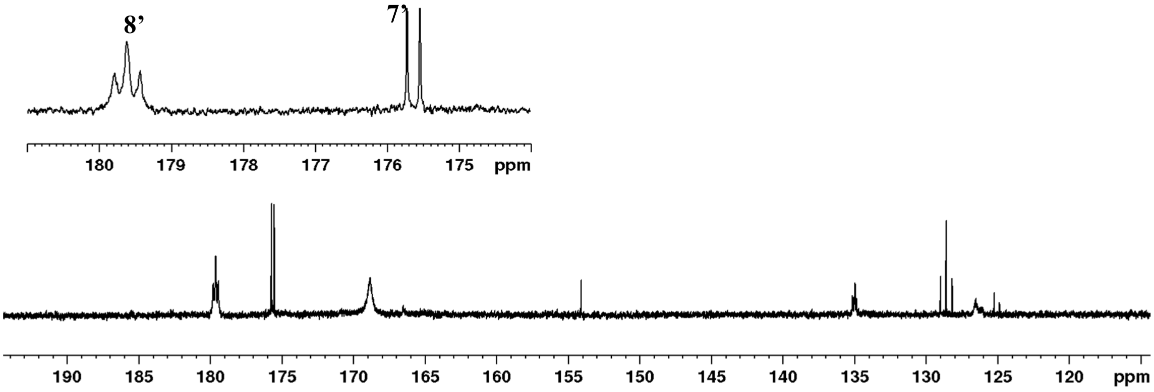

Figure S26. $^{13}C\{^{1}H\}$ NMR spectrum (75.4 MHz) of the mixture of complexes **7’** and **8’** in CD_2Cl_2.

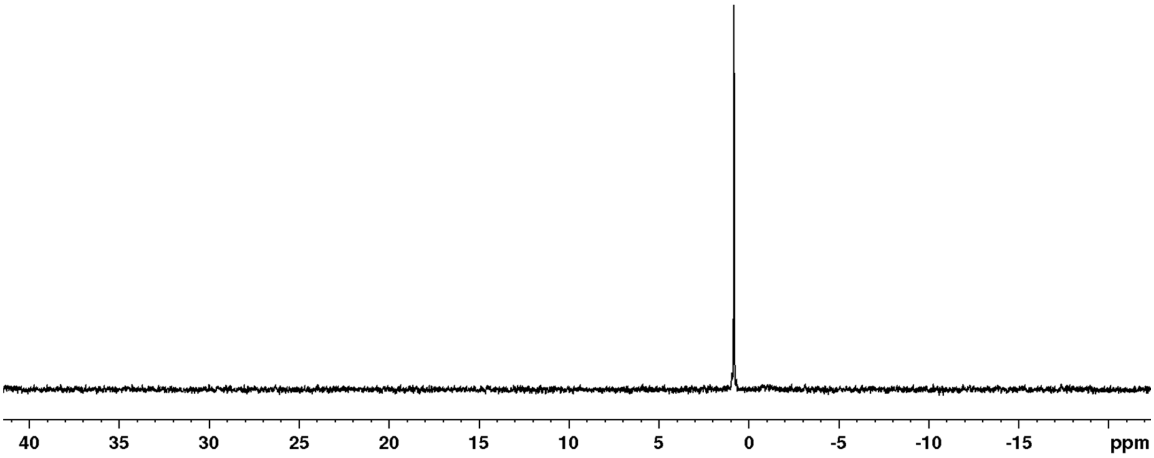

Figure S27. $^{31}P\{^{1}H\}$ NMR spectrum (121.5 MHz) of complex **8** in CD_2Cl_2.

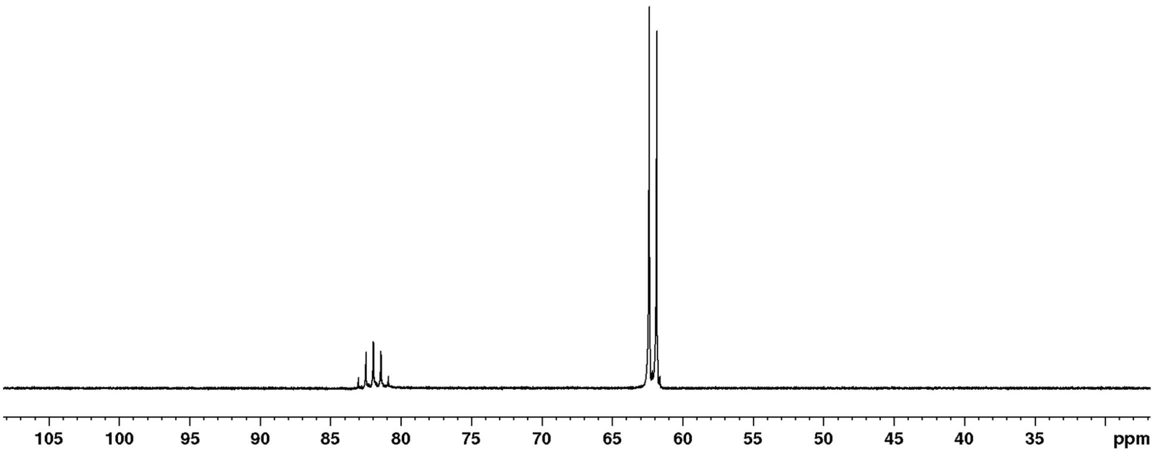

Figure S28. ^{19}F NMR spectrum (282.4 MHz) of complex **8** in CD_2Cl_2.

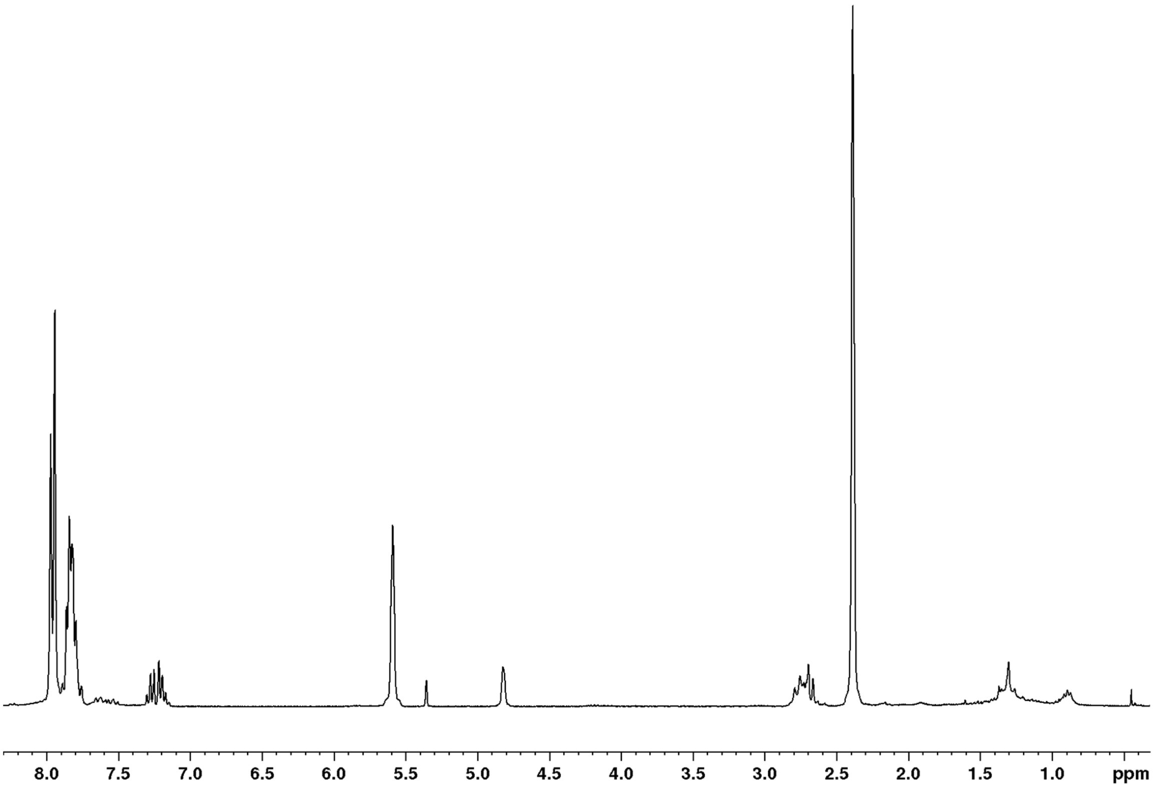

Figure S29. ^{1}H NMR spectrum (300.1 MHz) of complex **8** in CD_2Cl_2.

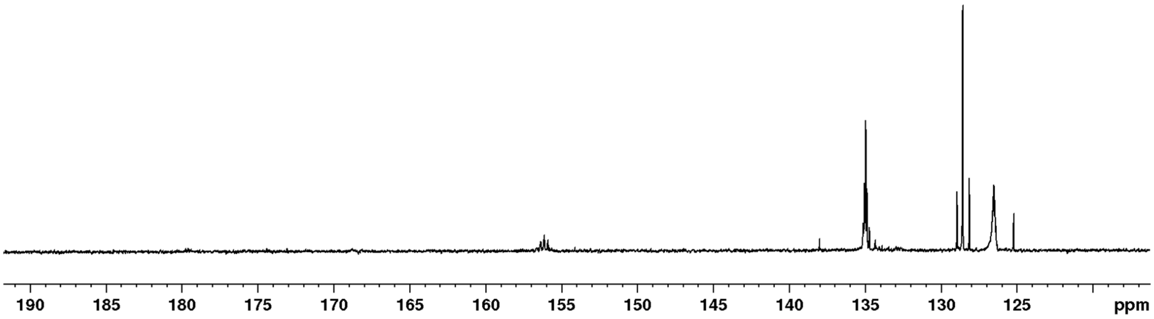

Figure S30. $^{13}C\{^{1}H\}$ NMR spectrum (75.4 MHz) of complex **8** in CD_2Cl_2.

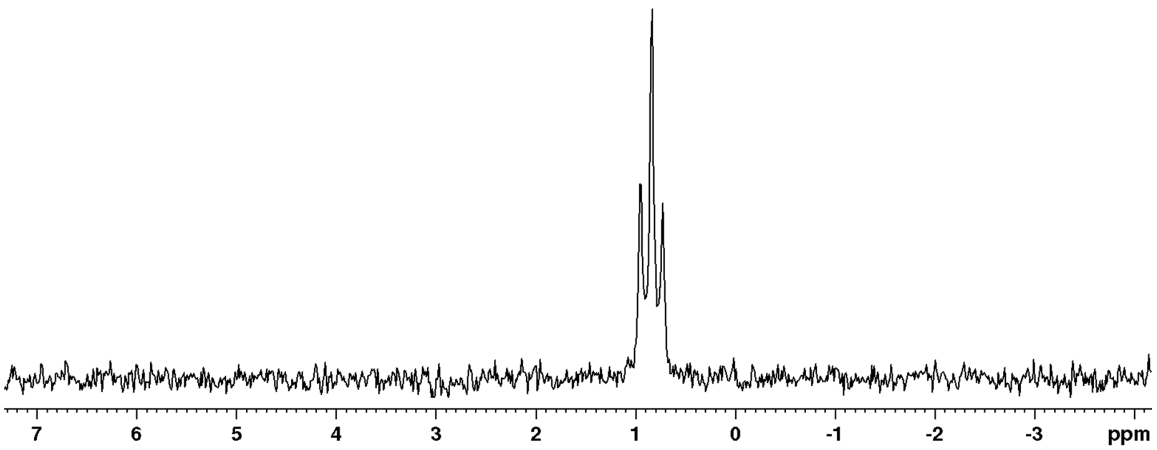

Figure S31. $^{31}P\{^1H\}$ NMR spectrum (121.5 MHz) of complex **8’** in CD_2Cl_2.

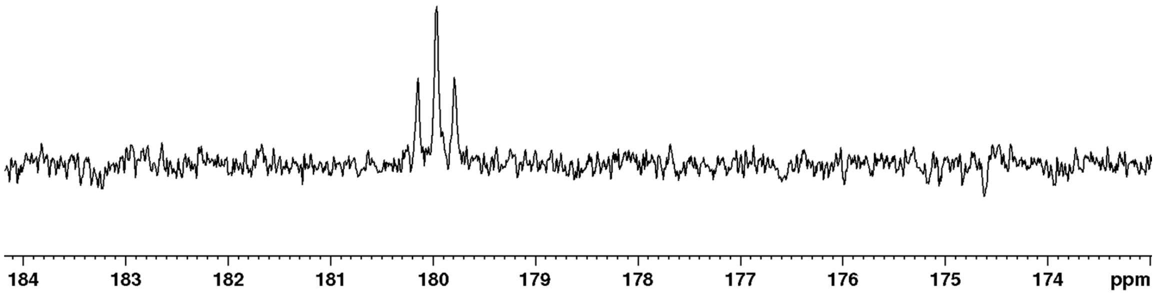

Figure S32. Section of the $^{13}C\{^1H\}$ NMR spectrum (75.4 MHz) of complex **8’** in CD_2Cl_2.

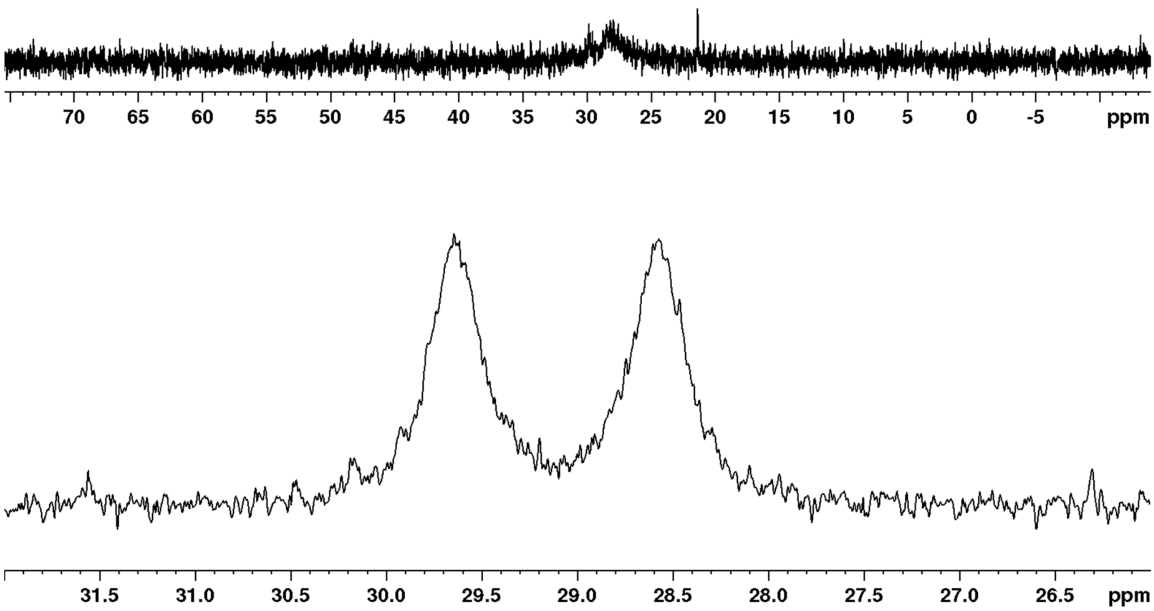

Figure S33. $^{31}P\{^{1}H\}$ NMR spectrum (121.5 MHz) of complex **9** in acetone-*d*^{6} at 243 K (bottom) and rt (top).

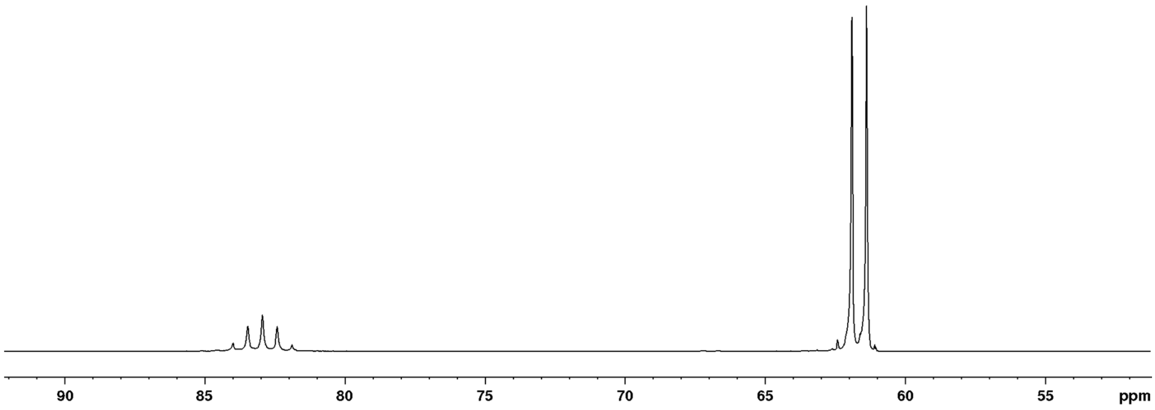

Figure S34. ^{19}F NMR spectrum (282.4 MHz) of complex **9** in acetone-*d*^{6}.

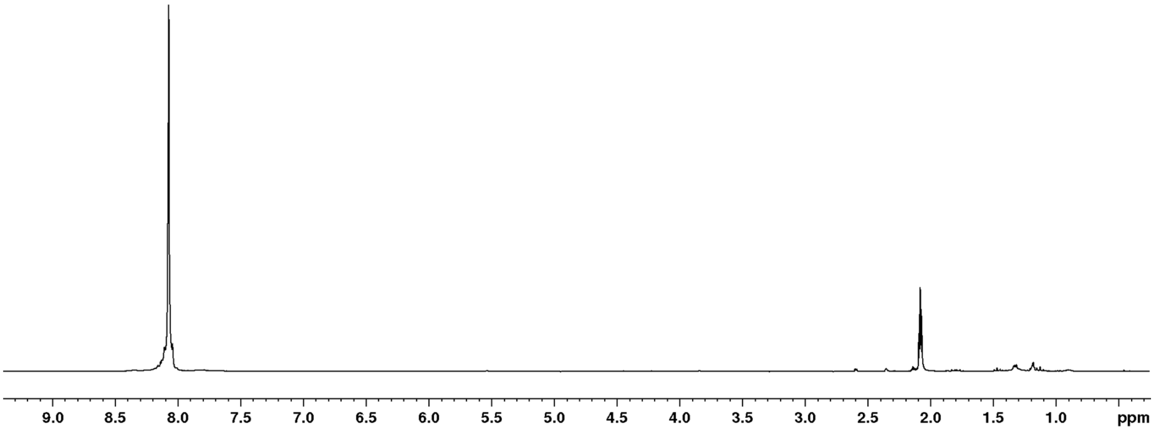

Figure S35. ^{1}H NMR spectrum (300.1 MHz) of complex **9** in acetone-d^{6}.

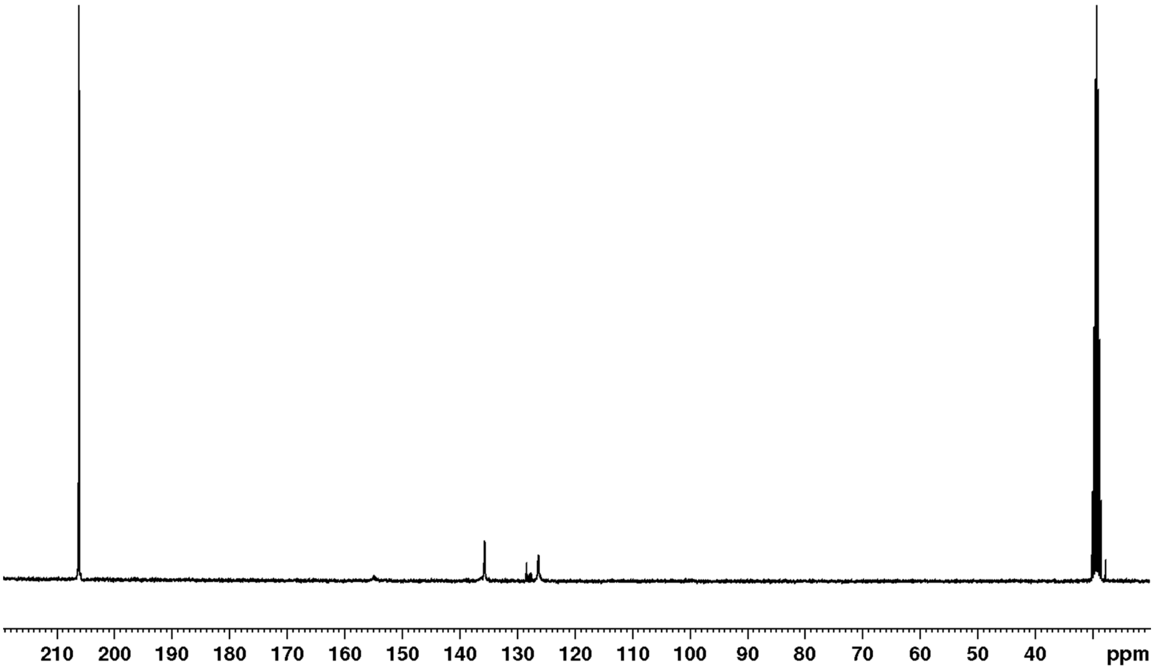

Figure S36. $^{13}C\{^{1}H\}$ NMR spectrum (75.4 MHz) of complex **9** in acetone-d^{6}.

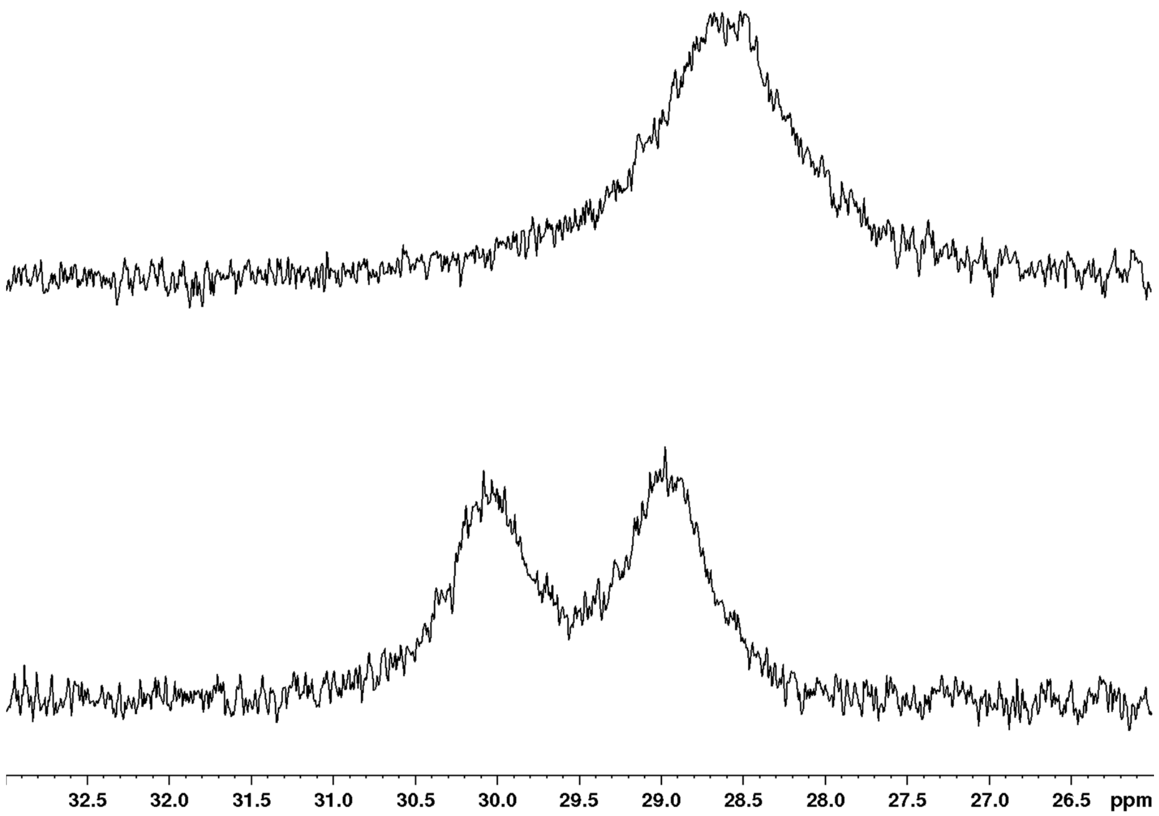

Figure S37. $^{31}P\{^{1}H\}$ NMR spectrum (121.5 MHz) of complex **9’** in acetone-*d*^{6} at 203 K (bottom) and rt (top).

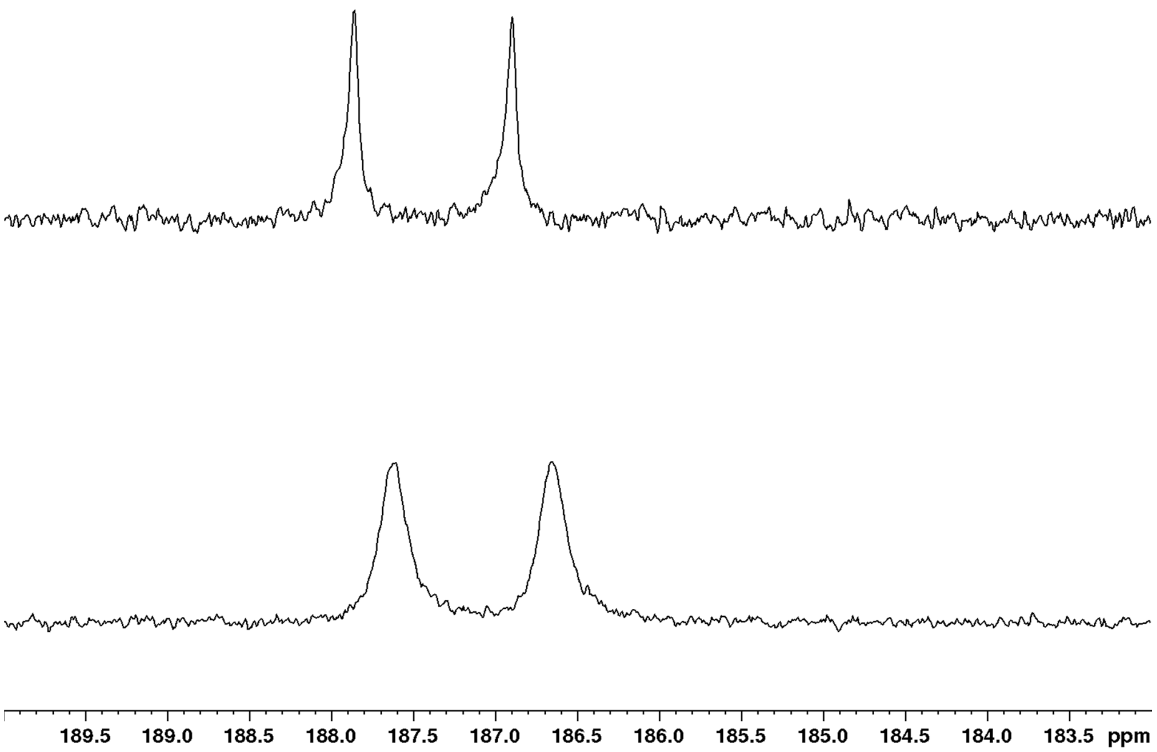

Figure S38. Section of the $^{13}C\{^1H\}$ NMR spectrum (75.4 MHz) of complex **9'** in acetone-d^6 at 203 K (bottom) and rt (top).

IR spectra

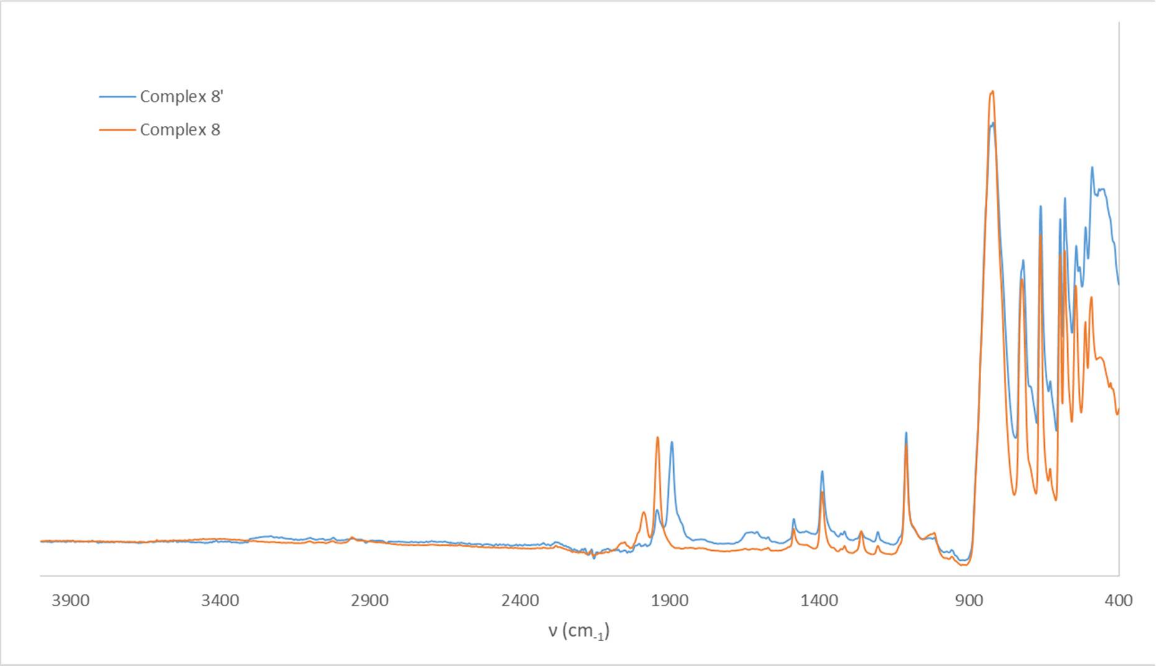

Figure S39. IR spectra of complexes **8** (orange) and **8’** (blue).

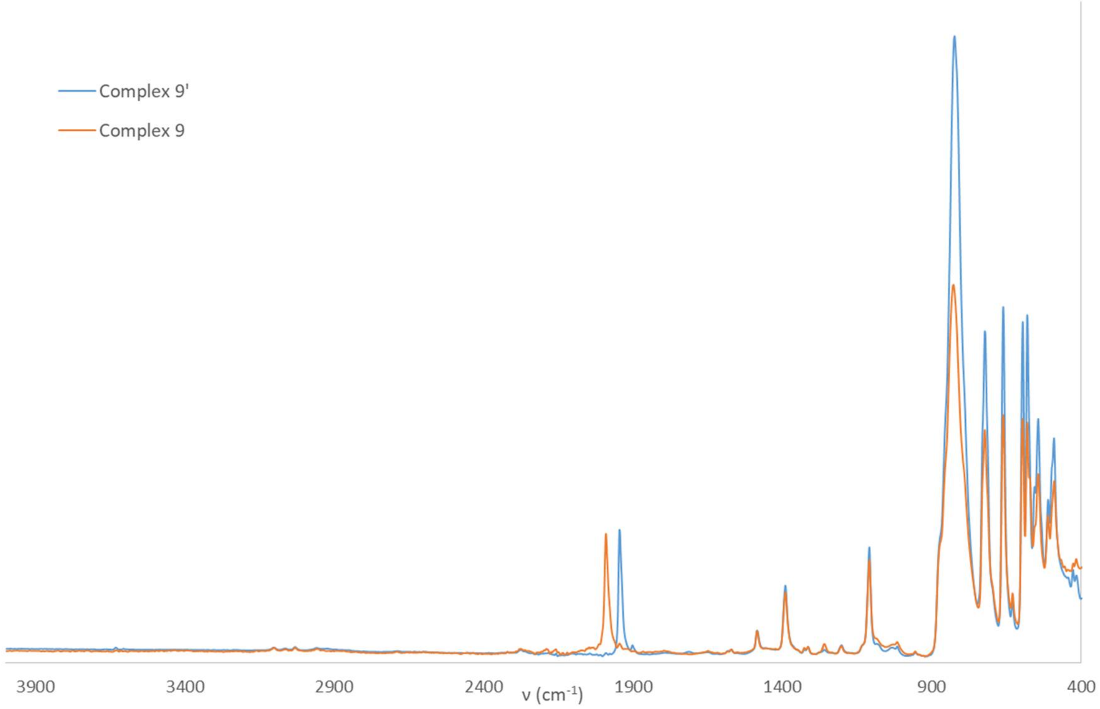

Figure S40. IR spectra of complexes **9** (orange) and **9’** (blue).

Computational details and xyz coordinates of the compounds under study

Calculations were run using the Gaussian 09 (Revision D.01) program package.[74] In the case of phosphines and phosphine radical cations the CAM-B3LYP functional was used and 6-311G(d,p) basis set were employed for all atoms. For the nickel complexes, the B3LYP functional was chosen. Nickel was described with RECPs and the associated LANL2DZ basis sets[75] while the ligands were described with 6-31G(d,p). All calculated structures were identified as minima (no negative eigenvalues).

Table S1: Cartesian Coordinates of optimized structures of PAr_3.

**Compound 1**			
P	0.00043	0.00002	2.06657
C	1.65336	0.04583	1.24954
C	-0.78655	-1.45426	1.24952
C	-0.86575	1.40879	1.24985
C	-2.06536	1.82579	1.82767
C	-0.38810	2.08703	0.13131
C	-2.78891	2.87775	1.29217
H	-2.44209	1.32634	2.71278
C	-2.28874	3.52791	0.17700
C	-1.09446	3.15116	-0.40959
H	0.54541	1.79291	-0.33032
H	-0.71570	3.67263	-1.27656
H	-3.71893	3.18974	1.74432
S	-3.22021	4.91200	-0.52830
C	2.00187	-0.70728	0.13125
C	2.61435	0.87632	1.82719
C	3.27662	-0.62777	-0.40970
H	1.28040	-1.36882	-0.33016
C	3.88716	0.97670	1.29171
C	4.20008	0.21817	0.17670
H	2.37020	1.45248	2.71219
H	4.62244	1.62611	1.74378
S	5.86461	0.33214	-0.52838
C	-0.54677	-2.70193	1.82633
C	-1.61424	-1.37928	0.13215
C	-1.09645	-3.85435	1.29094
C	-2.18303	-2.52294	-0.40864
C	-1.91110	-3.74583	0.17691
H	-0.90090	-4.81597	1.74234
S	-2.64484	-5.24424	-0.52817
H	0.07525	-2.77878	2.71064
F	-3.49211	5.55917	0.93427
F	5.86473	1.95189	-0.42652
F	-3.06662	-5.80459	0.93471
F	-3.29529	-6.57763	-1.15626
F	-1.24141	-6.05329	-0.42924
F	-4.09905	-4.54129	-0.67766
F	-1.88430	5.81932	-0.68090
F	-4.62267	4.10166	-0.42623
F	6.56077	0.24389	0.93429
F	7.34459	0.43564	-1.15644
H	3.53889	-1.21683	-1.27644
F	5.28569	0.42947	-2.04144
F	5.98186	-1.27852	-0.68083
F	-3.01485	4.36177	-2.04125
F	-4.05033	6.14152	-1.15657
F	-2.27391	-4.79012	-2.04151
H	-1.82709	-0.42357	-0.32864
H	-2.82525	-2.45540	-1.27467

**P(*p*-CF$_3$-C$_6$H$_4$)$_3$**			
P	0.00116	0.00005	1.82779
C	-0.73752	1.48047	1.01292
C	1.65237	-0.10085	1.01268
C	-0.91220	-1.37934	1.01267
C	-0.79643	-2.64026	1.59550
C	-1.71801	-1.23374	-0.11690
C	-1.44780	-3.73787	1.05414
H	-0.19254	-2.76747	2.48680
C	-2.24135	-3.57690	-0.07066
C	-2.38058	-2.32416	-0.65429
H	-1.83350	-0.26264	-0.58041
H	-3.00581	-2.20258	-1.53031
H	-1.34866	-4.71356	1.50999
C	-2.96931	-4.73967	-0.67626
C	-0.20295	2.11280	-0.10984
C	-1.89425	2.00291	1.58942
C	-0.81735	3.23123	-0.64704
H	0.70108	1.73342	-0.56827
C	-2.52049	3.11495	1.04807
C	-1.97879	3.72895	-0.07014
H	-2.31093	1.53789	2.47567
H	-3.42045	3.51077	1.49886
H	-0.39500	3.71771	-1.51773
C	-2.62364	4.93966	-0.67576
C	2.68519	0.63445	1.59255
C	1.93063	-0.87560	-0.11369
C	3.96140	0.61974	1.05118
C	3.20638	-0.90378	-0.65094
C	4.22025	-0.15217	-0.07048
H	4.75579	1.19708	1.50465
C	5.59117	-0.20020	-0.67621
H	1.14842	-1.46467	-0.57486
H	3.41487	-1.50943	-1.52439
H	2.49238	1.22434	2.48151
F	-3.73305	5.30913	-0.01999
F	-2.97058	4.72339	-1.95678
F	-1.79042	5.99433	-0.67752
F	6.46805	0.57074	-0.01724
F	5.57851	0.21385	-1.95562
F	6.08459	-1.45041	-0.68322
F	-2.74115	-5.88433	-0.01706
F	-2.60447	-4.93635	-1.95532
F	-4.29852	-4.54094	-0.68388

**P(*p*-F-C$_6$H$_4$)$_3$**			
P	-0.00132	-0.00014	1.40530
C	0.93107	-1.36671	0.59435
C	-1.65039	-0.12315	0.59314
C	0.71711	1.48930	0.59297
C	0.45701	2.72455	1.19021
C	1.51194	1.45876	-0.55193
C	0.95205	3.90135	0.65251
H	-0.14072	2.76725	2.09380
C	1.73182	3.82916	-0.48530
C	2.02564	2.62723	-1.09638
H	1.74107	0.51292	-1.02628
H	2.64755	2.61659	-1.98215
H	0.75460	4.86402	1.10577
F	2.22588	4.96390	-1.00809
C	0.50198	-2.05047	-0.54217
C	2.13791	-1.74804	1.18475
C	1.25865	-3.07917	-1.08530
H	-0.43694	-1.78488	-1.01113
C	2.91125	-2.76425	0.64793
C	2.45345	-3.41428	-0.48163
H	2.47829	-1.24341	2.08204
H	3.84925	-3.06568	1.09566
H	0.93434	-3.62071	-1.96455
F	3.19081	-4.40877	-1.00350
C	-2.59600	-0.95207	1.20028
C	-2.01524	0.56661	-0.56222
C	-3.86275	-1.11185	0.66251
H	-2.33882	-1.47977	2.11183
C	-3.28359	0.42684	-1.10719
C	-4.18375	-0.41478	-0.48581
H	-4.60242	-1.75332	1.12353
F	-5.41320	-0.55432	-1.00918
H	-1.30606	1.22745	-1.04440
H	-3.58060	0.95997	-2.00108

**P(*p*-Me-C$_6$H$_4$)$_3$**			
P	-0.00076	-0.00169	1.43441
C	1.31902	-0.99986	0.62390
C	-1.52512	-0.64388	0.62246
C	0.20509	1.64022	0.62373
C	-0.45097	2.72527	1.20236
C	0.98372	1.87255	-0.50956
C	-0.35209	3.99526	0.65381
H	-1.04691	2.57586	2.09646
C	0.42152	4.22832	-0.47848
C	1.08930	3.14578	-1.04693
H	1.51660	1.05245	-0.97494
H	1.70403	3.30171	-1.92765
H	-0.87699	4.82131	1.12145
C	0.54891	5.60610	-1.07057
C	1.13466	-1.78121	-0.51634
C	2.58371	-0.98188	1.20904
C	2.18549	-2.50732	-1.05429
H	0.16016	-1.82684	-0.98678
C	3.63519	-1.70073	0.66009
C	3.45415	-2.47758	-0.47938
H	2.74903	-0.39854	2.10859
H	4.61067	-1.66531	1.13296
H	2.01624	-3.11032	-1.94063
C	4.58493	-3.27400	-1.07271
C	-2.14561	-1.74548	1.20868
C	-2.10679	-0.09369	-0.51936
C	-3.29521	-2.29395	0.65967
C	-3.26226	-0.63794	-1.05740
C	-3.87493	-1.74858	-0.48097
H	-3.75552	-3.15412	1.13369
C	-5.13179	-2.32619	-1.07406
H	-1.65602	0.77112	-0.99058
H	-3.69779	-0.18988	-1.94477
H	-1.72533	-2.18002	2.10941
H	5.51569	-3.10754	-0.52961
H	4.75240	-3.00277	-2.11783
H	4.36853	-4.34491	-1.04574
H	-5.45820	-3.21173	-0.52773
H	-4.98009	-2.61194	-2.11784
H	-5.94763	-1.59922	-1.05152
H	-0.07096	6.32684	-0.53631
H	0.24511	5.61397	-2.12017
H	1.58279	5.95806	-1.02896

**P(*p*-OMe-C$_6$H$_4$)$_3$**			
P	0.00111	-0.00079	1.47415
C	-1.19388	-1.14410	0.66564
C	-0.39223	1.60610	0.66673
C	1.58922	-0.46317	0.66601
C	2.76575	-0.02579	1.26580
C	1.69767	-1.23538	-0.49447
C	4.01409	-0.31573	0.72896
H	2.71386	0.55708	2.17923
C	4.09906	-1.08060	-0.42962
C	2.93084	-1.54232	-1.03462
H	0.80332	-1.60761	-0.97893
H	3.02049	-2.14322	-1.93092
H	4.90245	0.04772	1.22611
O	5.26019	-1.43299	-1.03583
C	-1.91493	-0.85197	-0.49604
C	-1.40620	-2.38061	1.26642
C	-2.79808	-1.76555	-1.03646
H	-1.78798	0.10795	-0.98136
C	-2.28268	-3.31568	0.72973
C	-2.98551	-3.00697	-0.43008
H	-0.87687	-2.62697	2.18059
H	-2.41465	-4.26577	1.22808
H	-3.36180	-1.54282	-1.93368
O	-3.87237	-3.83522	-1.03614
C	-1.35719	2.40739	1.26818
C	0.21978	2.08477	-0.49562
C	-1.73086	3.63314	0.73111
C	-0.13166	3.30580	-1.03615
C	-1.11383	4.08763	-0.42956
H	-2.48815	4.22165	1.22960

**P(*m*-CF$_3$-C$_6$H$_3$)$_3$**			
P	-0.00073	0.00073	-1.68084
C	1.20431	1.13276	-0.86302
C	-1.58354	0.47720	-0.86223
C	0.37815	-1.60860	-0.86311
C	-0.14811	-2.75613	-1.46418
C	1.16406	-1.75019	0.27133
C	0.09228	-4.00451	-0.92302
H	-0.74591	-2.67331	-2.36333
C	0.87808	-4.14261	0.21612
C	1.41132	-3.01299	0.80310
H	1.59421	-0.88177	0.75412
C	2.27193	-3.11472	2.03003
C	-0.47549	-5.24305	-1.55538
H	1.07427	-5.12058	0.63455
C	0.93507	1.88365	0.27208
C	2.46088	1.25084	-1.46523
C	1.90586	2.72848	0.80349
H	-0.03177	1.82212	0.75575
C	3.42249	2.08277	-0.92459
C	3.15033	2.83168	0.21531
H	2.68727	0.69211	-2.36492
C	4.77837	2.21071	-1.55860
C	1.56500	3.52370	2.03142
H	3.89969	3.49019	0.63341
C	-2.31630	1.50434	-1.46505
C	-2.09708	-0.13061	0.27425
C	-3.51774	1.91997	-0.92371
C	-3.31426	0.28650	0.80643
C	-4.02807	1.31068	0.21758
C	-4.30896	3.02803	-1.55834

**P(3,4,5-F-C$_6$H$_2$)$_3$**			
P	0.00032	0.00030	-1.52611
C	1.61610	0.34042	-0.70679
C	-1.10238	1.22917	-0.70656
C	-0.51312	-1.56926	-0.70718
C	-1.55645	-2.27262	-1.30902
C	0.08768	-2.08521	0.43809
C	-1.99508	-3.45683	-0.75587
H	-2.03544	-1.91750	-2.21305
C	-1.40362	-3.97659	0.38317
C	-0.36008	-3.27977	0.96579
H	0.90654	-1.58177	0.93404
F	0.20897	-3.78911	2.05986
F	-2.99434	-4.13841	-1.31804
F	-1.82358	-5.12626	0.89982
C	1.76160	1.11446	0.44148
C	2.74760	-0.20699	-1.31132
C	3.01985	1.32423	0.96937
H	0.91556	1.56845	0.93956
C	3.99238	0.00550	-0.75801
C	4.14588	0.77338	0.38396
H	2.68014	-0.79585	-2.21766
F	5.08301	-0.51477	-1.32276
F	3.17561	2.06774	2.06627
F	5.35135	0.98485	0.90079
C	-1.19410	2.48297	-1.31066
C	-1.84553	0.96770	0.44158
C	-2.00068	3.45448	-0.75711
C	-2.65648	1.95223	0.96972
C	-2.74253	3.20299	0.38468
F	-2.09554	4.65926	-1.32150

**PPh$_3$**			
P	-0.00049	0.00043	-1.23116
C	-1.58415	0.48138	-0.41861
C	0.37450	-1.61173	-0.41874
C	1.20866	1.13093	-0.41896
C	2.47397	1.22599	-1.00118
C	0.93399	1.89915	0.71080
C	3.44759	2.04808	-0.45530
H	2.69606	0.65065	-1.89352
C	3.16377	2.80568	0.67296
C	1.90493	2.73233	1.25064
H	-0.04481	1.85070	1.17114
H	1.67482	3.32595	2.12763
H	4.42590	2.10586	-0.91789
H	3.92008	3.45615	1.09600
C	-2.10875	-0.13811	0.71399
C	-2.30224	1.52650	-1.00262
C	-3.31573	0.28511	1.25463
H	-1.57493	-0.95942	1.17569
C	-3.50118	1.95745	-0.45609
C	-4.01204	1.33534	0.67481
H	-1.91737	2.00473	-1.89683
H	-4.04288	2.77318	-0.92005
H	-3.71184	-0.20874	2.13414
H	-4.95350	1.66408	1.09853
C	-0.17799	-2.75497	-0.99887
C	1.18040	-1.75779	0.70878
C	0.04807	-4.00900	-0.45291
H	-0.78962	-2.65986	-1.88980
C	1.41749	-3.01515	1.24845
C	0.84936	-4.14189	0.67306

O	-1.39044	5.26876	-1.03643
H	0.98755	1.49523	-0.98156
H	0.34187	3.68282	-1.93397
H	-1.83398	2.07199	2.18302
C	6.47542	-0.99344	-0.46612
C	-2.37762	6.10196	-0.46546
C	-4.10388	-5.10564	-0.46414
H	7.26476	-1.38058	-1.10651
H	6.60898	-1.38579	0.54700
H	6.53491	0.09925	-0.44168
H	-2.43869	6.97827	-1.10684
H	-2.10226	6.41512	0.54667
H	-3.35361	5.60696	-0.43798
H	-4.83430	-5.59487	-1.10468
H	-4.51184	-5.02173	0.54828
H	-3.18915	-5.70631	-0.43692

H	-4.97315	1.62947	0.63625
H	-1.55851	-0.93562	0.75831
C	-3.83024	-0.40539	2.03584
H	-1.94732	1.97897	-2.36577
F	2.59217	4.27201	2.45126
F	1.20726	2.72062	3.04592
F	0.52962	4.34790	1.80512
F	4.89683	1.45263	-2.65646
F	5.74931	1.84390	-0.70767
F	5.03181	3.47962	-1.91448
F	2.40638	-4.37809	2.45089
F	1.75693	-2.40216	3.04457
F	3.50321	-2.63104	1.80167
F	-1.19113	-4.96751	-2.65344
F	-1.27879	-5.89890	-0.70364
F	0.49609	-6.09809	-1.91027
F	-4.99232	0.10871	2.45614
F	-2.95504	-0.31090	3.04944
F	-4.02444	-1.71472	1.81150
F	-3.71409	3.50847	-2.65796
F	-4.47663	4.05346	-0.70886
F	-5.53470	2.61102	-1.91139

F	-3.52859	4.14096	0.90173
H	-1.81550	0.00784	0.93934
F	-3.37825	1.71501	2.06653
H	-0.65030	2.71936	-2.21685

H	-0.39288	-4.88494	-0.91367
H	1.03543	-5.12195	1.09602
H	1.62986	-0.88601	1.16736
H	2.04923	-3.11258	2.12359

Table S2: Cartesian Coordinates of optimized structures of radical cations of PAr_3.

RC_Compound 1

P	0.00004	0.00080	1.12449
C	-0.04493	-1.72583	0.69082
C	-1.47288	0.90287	0.69090
C	1.51784	0.82499	0.69059
C	1.76765	2.09145	1.23567
C	2.46636	0.21460	-0.13700
C	2.94340	2.75158	0.93355
H	1.05338	2.56220	1.90041
C	3.86439	2.13541	0.10025
C	3.64224	0.87826	-0.43777
H	2.28644	-0.76313	-0.56385
H	4.37301	0.41993	-1.08829
H	3.14252	3.72816	1.35037
S	5.41620	3.00505	-0.29718
C	-1.04719	-2.24231	-0.13735
C	0.92672	-2.57521	1.23659
C	-1.05992	-3.59250	-0.43807
H	-1.80383	-1.59783	-0.56474
C	0.91098	-3.92355	0.93458
C	-0.08246	-4.41327	0.10059
H	1.69105	-2.19181	1.90178
H	1.65703	-4.58408	1.35197
S	-0.10455	-6.19198	-0.29707
C	-2.69435	0.48506	1.23578
C	-1.41919	2.02987	-0.13626
C	-3.85436	1.17248	0.93370
C	-2.58232	2.71565	-0.43699
C	-3.78190	2.27838	0.10063
H	-4.79950	0.85587	1.35029
S	-5.31167	3.18609	-0.29717
H	-2.74438	-0.36913	1.90025
F	5.61648	3.42557	1.25050
F	1.49031	-6.18458	-0.56526
F	-5.77677	3.14820	1.25025
F	-6.64926	3.97811	-0.64601
F	-6.10114	1.80083	-0.56809
F	-4.57778	4.60348	-0.04187
F	6.27539	1.65974	-0.04280
F	4.61271	4.38239	-0.56718
F	0.15777	-6.57582	1.25084
F	-0.12248	-7.74641	-0.64569
H	-1.82175	-3.99636	-1.08905
F	-0.36684	-5.87276	-1.86041
F	-1.69952	-6.26371	-0.04453
F	5.27192	2.61683	-1.86025
F	6.77172	3.76620	-0.64558
F	-4.90259	3.25620	-1.86000
H	-0.48268	2.36375	-0.56289
H	-2.55128	3.57791	-1.08724

RC_P(*p*-CF_3-C_6H_4)$_3$

P	-0.00042	-0.01766	1.02157
C	1.47688	-0.90929	0.58605
C	-1.51754	-0.83962	0.58245
C	0.04115	1.70930	0.58796
C	-0.91231	2.56162	1.15977
C	1.01993	2.22256	-0.27239
C	-0.90219	3.90868	0.85001
H	-1.65434	2.17805	1.84991
C	0.06116	4.40702	-0.01686
C	1.02111	3.57034	-0.57484
H	1.76335	1.57301	-0.71580
H	1.76879	3.97674	-1.24380
H	-1.63037	4.57653	1.29008
C	0.05947	5.86810	-0.39695
C	1.43177	-2.05543	-0.21764
C	2.69854	-0.46069	1.10693
C	2.60420	-2.72306	-0.51806
H	0.49355	-2.41322	-0.62033
C	3.86496	-1.13449	0.79803
C	3.81406	-2.25964	-0.01475
H	2.73881	0.40603	1.75568
H	4.81242	-0.79888	1.19748
H	2.58221	-3.60629	-1.14283
C	5.08867	-2.97323	-0.39690
C	-1.76733	-2.11324	1.11161
C	-2.47170	-0.21502	-0.23086
C	-2.94582	-2.76537	0.80189
C	-3.64718	-0.87538	-0.53203
C	-3.87920	-2.14624	-0.01930
H	-3.15110	-3.74649	1.20871
C	-5.14838	-2.86916	-0.40153
H	-2.29093	0.77061	-0.63912
H	-4.38868	-0.40464	-1.16384
H	-1.04999	-2.59024	1.76889
F	6.07022	-2.72111	0.47143
F	5.49690	-2.57399	-1.60670
F	4.90182	-4.29504	-0.44654
F	-5.44416	-3.83582	0.47096
F	-5.01410	-3.43091	-1.60885
F	-6.18279	-2.02485	-0.45842
F	-0.65372	6.59833	0.46305
F	-0.47064	6.02941	-1.61518
F	1.30436	6.35367	-0.42800

RC_P(*p*-F-C_6H_4)$_3$

P	0.00104	-0.00213	0.77825
C	1.58211	-0.67573	0.32871
C	-1.37496	-1.03281	0.32899
C	-0.20504	1.70489	0.33031
C	-1.28126	2.41860	0.88270
C	0.70415	2.35873	-0.51395
C	-1.46207	3.75052	0.57594
H	-1.97463	1.93199	1.55818
C	-0.55299	4.36637	-0.27148
C	0.52639	3.69259	-0.82089
H	1.53835	1.82104	-0.94503
H	1.20298	4.21971	-1.48042
H	-2.28197	4.32458	0.98644
F	-0.72171	5.64687	-0.56432
C	1.69333	-1.80306	-0.49818
C	2.73930	-0.08741	0.86597
C	2.93754	-2.31559	-0.80430
H	0.80981	-2.26706	-0.91688
C	3.98333	-0.59638	0.55939
C	4.06168	-1.70460	-0.27109
H	2.66515	0.76648	1.52874
H	4.89109	-0.16359	0.95800
H	3.05531	-3.17538	-1.45033
F	5.25509	-2.19897	-0.56278
C	-1.45496	-2.32410	0.87608
C	-2.39798	-0.56689	-0.50942
C	-2.51998	-3.14384	0.56826
H	-0.68511	-2.68558	1.54769
C	-3.46614	-1.38483	-0.81720
C	-3.51013	-2.65903	-0.27360
H	-2.60727	-4.14273	0.97454
F	-4.53688	-3.44241	-0.56669
H	-2.34931	0.42657	-0.93537
H	-4.26253	-1.05809	-1.47258

RC_P(*p*-Me-C_6H_4)$_3$

P	-0.00019	0.00059	0.78215
C	1.43839	0.94496	0.33933
C	0.09832	-1.71726	0.33844
C	-1.53756	0.77419	0.33993
C	-2.72914	0.26345	0.87167
C	-1.58187	1.90795	-0.48347
C	-3.93506	0.86010	0.56101
H	-2.71187	-0.59724	1.53031
C	-3.99185	1.98283	-0.26620
C	-2.79629	2.49150	-0.78104
H	-0.67142	2.31667	-0.90244
H	-2.82258	3.35796	-1.43153
H	-4.85064	0.45517	0.97470
C	-5.29845	2.64460	-0.58852
C	2.44498	0.41353	-0.47901
C	1.59036	2.23449	0.86666
C	3.55879	1.17213	-0.77534
H	2.34592	-0.58080	-0.89478
C	2.71140	2.97899	0.55747
C	3.71476	2.46363	-0.26459
H	0.83464	2.65237	1.52167
H	2.81773	3.97555	0.96846
H	4.32421	0.75928	-1.42204
C	4.94189	3.26331	-0.58655
C	1.14216	-2.49179	0.86233
C	-0.86832	-2.32530	-0.47478
C	1.22823	-3.83468	0.55267
C	-0.76682	-3.66908	-0.77110
C	0.27777	-4.44765	-0.26499
H	2.04142	-4.42343	0.95950
C	0.35793	-5.91025	-0.58683
H	-1.68230	-1.74323	-0.88743
H	-1.50891	-4.12750	-1.41428
H	1.88356	-2.04405	1.51399
H	4.86054	4.28836	-0.22750
H	5.11944	3.28920	-1.66342
H	5.82364	2.81255	-0.12392
H	1.28443	-6.35241	-0.22312
H	0.29729	-6.07670	-1.66414
H	-0.47581	-6.44844	-0.12876
H	-6.14509	2.05985	-0.23142
H	-5.40859	2.78765	-1.66515
H	-5.35072	3.63241	-0.12349

RC_P(*p*-OMe-C_6H_4)$_3$

P	0.00093	-0.00622	0.82548
C	-0.07091	1.70547	0.36230
C	1.52009	-0.79646	0.36129
C	-1.44307	-0.92254	0.35322
C	-1.63088	-2.21018	0.87329
C	-2.43839	-0.37320	-0.47573
C	-2.75522	-2.94693	0.56093
H	-0.88931	-2.64342	1.53474
C	-3.73317	-2.39432	-0.27374
C	-3.55880	-1.10039	-0.78764
H	-2.31647	0.62016	-0.88765
H	-4.32748	-0.70206	-1.43631
H	-2.87185	-3.93850	0.97327
O	-4.85645	-3.01182	-0.63459
C	0.88257	2.28971	-0.49149
C	-1.07811	2.51374	0.90555
C	0.80551	3.62279	-0.80649
H	1.67157	1.68587	-0.92068
C	-1.16175	3.85497	0.59029
C	-0.21646	4.42199	-0.27218
H	-1.80682	2.09071	1.58738
H	-1.95070	4.45357	1.02157
H	1.51885	4.08726	-1.47491
O	-0.20135	5.70130	-0.64285
C	2.72403	-0.31618	0.89420
C	1.55105	-1.92449	-0.47894
C	3.92854	-0.91341	0.58195
C	2.74529	-2.52269	-0.79076
C	3.94839	-2.02586	-0.26722
H	4.84159	-0.51974	1.00439
O	5.05017	-2.67729	-0.63499
H	0.63392	-2.31415	-0.90090

RC_P(*m*-CF_3-C_6H_3)$_3$

P	-0.00105	-0.00145	-0.93383
C	-1.55689	-0.74703	-0.49249
C	1.42193	-0.97573	-0.48910
C	0.13062	1.71917	-0.49347
C	1.19098	2.46775	-1.02850
C	-0.82028	2.33224	0.32271
C	1.30042	3.80537	-0.71483
H	1.92009	2.01672	-1.69013
C	0.35863	4.41952	0.10785
C	-0.69304	3.68068	0.62042
H	-1.64659	1.77081	0.73953
C	-1.72630	4.31267	1.52182
C	2.44916	4.62681	-1.24895
H	0.44248	5.47379	0.33990
C	-1.61264	-1.86952	0.33386
C	-2.73466	-0.20970	-1.03551
C	-2.84395	-2.43281	0.63336
H	-0.71365	-2.29879	0.75725
C	-3.94750	-0.78373	-0.72122
C	-4.00892	-1.89881	0.11182
H	-2.70824	0.64227	-1.70346
C	-5.23281	-0.21116	-1.26874
C	-2.87558	-3.63210	1.54984
H	-4.96368	-2.35268	0.34604
C	1.54276	-2.26677	-1.02754
C	2.42712	-0.45901	0.32836
C	2.64949	-3.02757	-0.71910
C	3.53297	-1.24183	0.62361
C	3.65162	-2.51890	0.10406
C	2.79487	-4.42832	-1.26431
H	4.52535	-3.11621	0.33201
H	2.35214	0.53608	0.74789

RC_P(3,4,5-F-C_6H_2)$_3$

P	-0.00070	0.00155	-0.84084
C	-1.11385	1.31169	-0.38682
C	-0.57801	-1.61855	-0.38753
C	1.69140	0.30965	-0.38842
C	2.68162	-0.51907	-0.93464
C	2.03634	1.38312	0.44009
C	4.00010	-0.27973	-0.62134
H	2.45329	-1.33836	-1.60490
C	4.35721	0.77684	0.20978
C	3.36509	1.59785	0.73258
H	1.29972	2.04094	0.88030
F	3.72365	2.59180	1.52651
F	4.95819	-1.04469	-1.11401
F	5.61835	0.99666	0.49587
C	-2.22818	1.06648	0.42326
C	-0.88189	2.58934	-0.91563
C	-3.08064	2.10802	0.71580
H	-2.43728	0.09523	0.84996
C	-1.75064	3.60953	-0.60264
C	-2.85629	3.38315	0.21033
H	-0.04864	2.80730	-1.57153
F	-1.55776	4.82702	-1.07814
F	-4.13158	1.91468	1.49363
F	-3.67923	4.36373	0.49629
C	-1.78849	-2.06628	-0.93488
C	0.18012	-2.45002	0.44411
C	-2.23796	-3.32800	-0.61887
C	-0.29578	-3.70842	0.73945
C	-1.50078	-4.16153	0.21559
F	-3.37765	-3.77922	-1.11226
F	-1.93858	-5.36387	0.50416
H	1.11674	-2.13744	0.88477

RC_PPh_3

P	-0.00001	-0.00007	-0.66293
C	1.72430	-0.03663	-0.22674
C	-0.83048	1.51153	-0.22684
C	-0.89387	-1.47502	-0.22673
C	-2.17600	-1.66203	-0.76424
C	-0.32612	-2.45628	0.59556
C	-2.88653	-2.80713	-0.45751
H	-2.61017	-0.91821	-1.42190
C	-2.32412	-3.77368	0.36890
C	-1.04936	-3.59774	0.89289
H	0.66301	-2.31899	1.01254
H	-0.61892	-4.35174	1.53932
H	-3.87638	-2.95291	-0.87030
H	-2.88234	-4.67159	0.60290
C	2.28982	0.94485	0.59682
C	2.52771	-1.05254	-0.76543
C	3.63996	0.88930	0.89423
H	1.67609	1.73215	1.01466
C	3.87465	-1.09525	-0.45865
C	4.43010	-0.12579	0.36904
H	2.10095	-1.79976	-1.42410
H	4.49613	-1.87886	-0.87238
H	4.07742	1.63842	1.54162
H	5.48682	-0.16020	0.60308
C	-0.35164	2.71529	-0.76482
C	-1.96385	1.51063	0.59585
C	-0.98799	3.90323	-0.45812
H	0.50940	2.71928	-1.42277
C	-2.59068	2.70775	0.89317
C	-2.10590	3.89960	0.36874
H	-0.61951	4.83327	-0.87128
H	-2.60436	4.83203	0.60271

H	2.79189	-3.37870	-1.44962
H	2.72107	0.53664	1.56489
C	-5.13829	-4.31623	-0.13407
C	6.31937	-2.25300	-0.14449
C	-1.18992	6.59528	-0.13822
H	-6.09570	-4.59330	-0.56564
H	-5.21821	-4.30555	0.95583
H	-4.37420	-5.03089	-0.44974
H	7.04405	-2.92974	-0.58813
H	6.36303	-2.33353	0.94452
H	6.53669	-1.22852	-0.45666
H	-0.96756	7.55896	-0.58745
H	-1.12370	6.67517	0.94963
H	-2.19273	6.27720	-0.43363

C	4.59223	-0.66602	1.53223
H	0.78705	-2.67344	-1.68771
F	-4.10683	-4.11851	1.68379
F	-2.41741	-3.30286	2.76304
F	-2.08580	-4.60290	1.07394
F	-4.99197	0.74398	-2.17320
F	-5.96145	0.32377	-0.28562
F	-5.96427	-1.16351	-1.84886
F	-1.52497	5.62035	1.66186
F	-1.69459	3.74817	2.73443
F	-2.95497	4.12395	1.02483
F	3.13585	3.95364	-2.17831
F	3.29152	4.94680	-0.26320
F	2.00610	5.76081	-1.79329
F	5.64255	-1.47600	1.63976
F	4.09350	-0.45730	2.75598
F	5.01375	0.51548	1.06506
F	1.84458	-4.69926	-2.16519
F	2.69955	-5.32492	-0.27938
F	3.98370	-4.58592	-1.84796

F	0.38620	-4.51203	1.53689
H	-2.38404	-1.46218	-1.60776

H	-2.33942	0.58545	1.01311
H	-3.45865	2.71210	1.53992

Table S3: Cartesian Coordinates of optimized structures of $Ni(CO)_3PAr_3$.

$Ni(CO)_3P(p\text{-}SF_5\text{-}C_6H_4)_3$

P	-0.00050	0.00013	1.18394
C	-1.66161	0.19878	0.39376
C	1.00307	1.33932	0.39493
C	0.65835	-1.53828	0.39509
C	1.75297	-2.16854	1.00731
C	0.11444	-2.10469	-0.76655
C	2.30749	-3.32714	0.46740
H	2.17362	-1.75737	1.91973
C	1.74638	-3.86517	-0.68886
C	0.65263	-3.27082	-1.31210
H	-0.73733	-1.64403	-1.25502
H	0.22318	-3.70270	-2.20698
H	3.15345	-3.80282	0.94661
S	2.45896	-5.38799	-1.40069
C	-1.88040	0.95662	-0.76548
C	-2.75437	-0.43743	1.00319
C	-3.15927	1.07400	-1.31129
H	-1.05593	1.46654	-1.25183
C	-4.03481	-0.33806	0.46292
C	-4.22046	0.42068	-0.69081
H	-2.60847	-1.01015	1.91380
H	-4.86943	-0.83537	0.94005
S	-5.89551	0.56608	-1.40253
C	1.00321	2.60212	1.00774
C	1.76464	1.15131	-0.76730
C	1.73001	3.66124	0.46789
C	2.50614	2.19999	-1.31277
C	2.47562	3.44411	-0.68888
H	1.72021	4.63147	0.94760
S	3.43952	4.82184	-1.40045
H	0.43755	2.76082	1.92062
F	2.55517	-6.10013	0.06111
F	-6.17039	-1.03133	-1.23182
F	4.00855	5.26042	0.06144
F	4.29202	6.04427	-2.03338
F	2.19628	5.86072	-1.22305
F	4.74509	3.87364	-1.62633
F	0.98564	-6.04603	-1.62645
F	3.97973	-4.82918	-1.22346
F	-6.56096	0.83221	0.06023
F	-7.38044	0.69273	-2.03552
H	-3.31874	1.66474	-2.20423
F	-5.33977	0.30854	-2.91351
F	-5.73034	2.17209	-1.62173
F	2.41021	-4.77510	-2.91072
F	3.09267	-6.73678	-2.03384
F	2.93283	4.47400	-2.91058
H	1.79037	0.18353	-1.25624
H	3.09420	2.04386	-2.20806
Ni	-0.00252	0.00020	3.49828
C	-1.24966	1.22576	4.03697
C	-0.44132	-1.69195	4.03805
C	1.68150	0.46676	4.04034
O	-0.71587	-2.74481	4.40521
O	-2.02490	1.98951	4.40350
O	2.73001	0.75565	4.40892

$Ni(CO)_3P(p\text{-}CF_3\text{-}C_6H_4)_3$

P	0.00035	-0.00014	0.75578
C	1.67416	-0.02377	-0.03058
C	-0.85792	-1.43795	-0.02935
C	-0.81691	1.46170	-0.02924
C	-1.97082	1.97529	0.58482
C	-0.33526	2.08248	-1.19145
C	-2.63912	3.06938	0.04129
H	-2.34203	1.52235	1.49901
C	-2.15029	3.67654	-1.11846
C	-0.99653	3.18499	-1.73201
H	0.56186	1.71269	-1.67620
H	-0.61167	3.66734	-2.62409
H	-3.52668	3.46254	0.52508
C	-2.89766	4.83003	-1.73570
C	1.96925	-0.74912	-1.19447
C	2.69731	0.71584	0.58472
C	3.25446	-0.72835	-1.73570
H	1.19943	-1.33922	-1.67997
C	3.97874	0.74679	0.04072
C	4.25845	0.02210	-1.12092
H	2.49183	1.26163	1.50049
H	4.76412	1.31641	0.52563
H	3.47858	-1.30120	-2.62910
C	5.63115	0.09256	-1.73804
C	-0.72928	-2.69363	0.58643
C	-1.63386	-1.33107	-1.19321
C	-1.34358	-3.81889	0.04295
C	-2.25860	-2.45459	-1.73405
C	-2.11090	-3.69908	-1.11880
H	-1.24331	-4.78372	0.52819
C	-2.73636	-4.92333	-1.73533
H	-1.76000	-0.36949	-1.67906
H	-2.86668	-2.36252	-2.62754
H	-0.15379	-2.78845	1.50215
F	6.59096	0.26981	-0.80559
F	5.72684	1.12644	-2.60553
F	5.92973	-1.02958	-2.42691
F	-3.06177	-5.84318	-0.80249
F	-1.88933	-5.52299	-2.60341
F	-3.85802	-4.62114	-2.42337
F	-3.54584	5.56249	-0.80541
F	-3.82835	4.39654	-2.61691
F	-2.07215	5.65914	-2.40962
Ni	0.00180	0.00047	3.07534
C	0.26832	1.72846	3.60886
C	1.36590	-1.09358	3.60838
C	-1.62731	-0.63321	3.61007
O	0.43657	2.80641	3.96900
O	2.21618	-1.77763	3.96767
O	-2.64474	-1.02689	3.97043

$Ni(CO)_3P(p\text{-}Me\text{-}C_6H_4)_3$

P	-0.00023	-0.00064	0.14183
C	-1.51272	-0.72140	-0.63202
C	0.12966	1.67091	-0.62945
C	1.38120	-0.94735	-0.63365
C	2.64357	-0.90698	-0.01962
C	1.23553	-1.70454	-1.80475
C	3.72557	-1.59037	-0.56881
H	2.77772	-0.33841	0.89614
C	3.58392	-2.35130	-1.73812
C	2.32238	-2.39429	-2.34407
H	0.27086	-1.76002	-2.29893
H	2.18547	-2.97666	-3.25218
H	4.69406	-1.53927	-0.07711
C	4.74989	-3.12466	-2.30569
C	-2.09158	-0.21964	-1.80653
C	-2.11466	-1.82936	-0.01385
C	-3.23384	-0.81367	-2.34506
H	-1.65288	0.63962	-2.30370
C	-3.24924	-2.42239	-0.56214
C	-3.83324	-1.92225	-1.73470
H	-1.69263	-2.22733	0.90448
H	-3.69386	-3.28238	-0.06707
H	-3.66657	-0.40630	-3.25575
C	-5.08811	-2.54199	-2.30123
C	-0.53199	2.74385	-0.01054
C	0.85452	1.92467	-1.80271
C	-0.48034	4.02385	-0.55688
C	0.90916	3.21187	-2.33922
C	0.24574	4.28291	-1.72814
H	-1.00528	4.83698	-0.06128
C	0.33414	5.68054	-2.29251
H	1.38136	1.11701	-2.30078
H	1.47896	3.38546	-3.24903
H	-1.08847	2.57489	0.90686
H	-5.09643	-3.62754	-2.16242
H	-5.19037	-2.33443	-3.37032
H	-5.98219	-2.14547	-1.80401
H	-0.60208	6.22942	-2.15117
H	0.56317	5.66728	-3.36204
H	1.12495	6.25649	-1.79577
H	5.69028	-2.57821	-2.18356
H	4.61266	-3.33262	-3.37075
H	4.86800	-4.08925	-1.79635
Ni	0.00160	-0.00432	2.47135
C	0.49814	-1.68255	2.98726
C	-1.69812	0.40410	2.99371
C	1.20667	1.26292	2.99179
O	0.80707	-2.73687	3.32783
O	1.96535	2.05640	3.33504
O	-2.76436	0.66348	3.33847

$Ni(CO)_3P(m\text{-}CF_3\text{-}C_6H_3)_3$

P	0.00065	-0.00056	0.86947
C	-0.96307	-1.36815	0.07862
C	-0.70290	1.51841	0.08062
C	1.66748	-0.15081	0.07971
C	2.74541	0.47804	0.71904
C	1.90429	-0.86844	-1.09959
C	4.02926	0.40428	0.17895
H	2.58732	1.01332	1.64932
C	4.26182	-0.31271	-0.99508
C	3.19531	-0.94983	-1.62765
H	1.09180	-1.36755	-1.61513
C	3.44381	-1.78626	-2.85893
C	5.16261	1.14359	0.84728
H	5.26163	-0.37901	-1.40731
C	-1.70412	-1.21296	-1.09974
C	-0.95651	-2.61694	0.71625
C	-2.42059	-2.28971	-1.62849
H	-1.73061	-0.25903	-1.61398
C	-1.66268	-3.69127	0.17540
C	-2.40125	-3.53273	-0.99760
H	-0.41281	-2.74881	1.64574
C	-1.58825	-5.04313	0.84220
C	-3.27047	-2.08493	-2.85860
H	-2.95884	-4.36501	-1.41029
C	-1.79035	2.13317	0.71766
C	-0.19674	2.08679	-1.09505
C	-2.36894	3.28208	0.17874
C	-0.77236	3.24593	-1.62189
C	-1.86103	3.84672	-0.99170
C	-3.57964	3.88974	0.84407
H	-2.30398	4.74595	-1.40291
H	0.64439	1.63582	-1.60901
C	-0.16883	3.88348	-2.84935
H	-2.17752	1.72494	1.64525
F	-3.41404	-3.22776	-3.55944
F	-2.73321	-1.15920	-3.68143
F	-4.50829	-1.65630	-2.53127
F	-1.45576	-4.92792	2.17986
F	-0.52685	-5.74868	0.39526
F	-2.69085	-5.77947	0.59680
F	4.50337	-1.33551	-3.56040
F	2.37263	-1.78594	-3.68064
F	3.69551	-3.07220	-2.53326
F	4.99429	1.20178	2.18459
F	5.24556	2.41517	0.39970
F	6.35120	0.55504	0.60427
F	-1.08571	4.58239	-3.54840
F	0.36406	2.95777	-3.67507
F	0.82185	4.73911	-2.51842
F	-3.55344	3.70707	2.18050
F	-4.71969	3.32841	0.38657
F	-3.66299	5.21477	0.60835
Ni	0.00014	-0.00132	3.17731
C	1.26786	-1.20353	3.72566
C	0.40688	1.69775	3.72584
C	-1.67500	-0.49842	3.72515
O	2.05474	-1.95033	4.10081
O	0.65982	2.75271	4.10096
O	-2.71529	-0.80649	4.10000

$Ni(CO)_3PPh_3$

P	0.13304	0.00058	0.00045
C	0.91088	1.64627	0.31788
C	0.90920	-1.09832	1.26699
C	0.90930	-0.54838	-1.58395
C	0.29108	-1.59064	-2.29403
C	2.07759	0.02704	-2.10652
C	0.83649	-2.05699	-3.48975
H	-0.62432	-2.03198	-1.91082
C	1.99997	-1.47821	-4.00135
C	2.61663	-0.43505	-3.30919
H	2.56509	0.83991	-1.57822
H	3.51862	0.02340	-3.70475
H	0.34703	-2.86486	-4.02603
H	2.41973	-1.83436	-4.93773
C	2.08166	1.80921	1.07407
C	0.29108	2.78360	-0.22526
C	2.62161	3.08123	1.27620
H	2.57021	0.94417	1.51063
C	0.83733	4.05179	-0.03024
C	2.00330	4.20356	0.72332
H	-0.62617	2.67357	-0.79635
H	0.34664	4.92120	-0.45823
H	3.52553	3.19314	1.86836
H	2.42376	5.19214	0.88387

C	0.28782	-1.19837	2.52256			
C	2.08074	-1.83371	1.03073			
C	0.83312	-2.00236	3.52307			
H	-0.62997	-0.64950	2.71231			
C	2.61980	-2.64570	2.03097			
C	1.99979	-2.72995	3.27843			
H	0.34114	-2.06787	4.48923			
H	2.41951	-3.36402	4.05408			
H	2.57044	-1.77769	0.06398			
H	3.52432	-3.21371	1.83236			
Ni	-2.19503	0.00120	-0.00014			
C	-2.71839	-1.71832	0.31727			
C	-2.71791	1.13667	1.32993			
C	-2.71665	0.58592	-1.64868			
O	-3.06597	-2.79683	0.51325			
O	-3.06502	1.84649	2.16540			
O	-3.06313	0.95512	-2.68116			

References

74. Frisch, M.J.; Schlegel, H.B.; Scuseria, G.E.; Robb, M.A.; Cheeseman, J.R.; Scalmani, G.; Barone, V.; Petersson, G.A.; Nakatsuji, H.; Li, X.; et al. *Gaussian 09, Revision D.01*; Gaussian, Inc.: Wallingford, CT, USA, 2016.
75. Hay, P.J.; Wadt, W.R. Ab initio effective core potentials for molecular calculations. Potentials for K to Au including the outermost core orbitals. *J. Chem. Phys.* **1985**, *82*, 299–310.
